# Differentiation of Human Umbilical Cord Matrix Mesenchymal Stem Cells into Neural-Like Progenitor Cells and Maturation into an Oligodendroglial-Like Lineage

**DOI:** 10.1371/journal.pone.0111059

**Published:** 2014-10-30

**Authors:** Cristiana Leite, N. Tatiana Silva, Sandrine Mendes, Andreia Ribeiro, Joana Paes de Faria, Tânia Lourenço, Francisco dos Santos, Pedro Z. Andrade, Carla M. P. Cardoso, Margarida Vieira, Artur Paiva, Cláudia L. da Silva, Joaquim M. S. Cabral, João B. Relvas, Mário Grãos

**Affiliations:** 1 Biocant - Technology Transfer Association, Biocant Park, Cantanhede, Portugal; 2 Blood and Transplantation Center of Coimbra, Portuguese Institute of the Blood and Transplantation, Coimbra, Portugal; 3 Instituto de Biologia Molecular e Celular, Porto, Portugal; 4 Institute for Biotechnology and Bioengineering and Department of Bioengineering, Instituto Superior Técnico, Universidade de Lisboa, Lisboa, Portugal; 5 Crioestaminal Saúde e Tecnologia, S.A., Biocant Park, Cantanhede, Portugal; Universidade Federal do Rio de Janeiro, Brazil

## Abstract

Mesenchymal stem cells (MSCs) are viewed as safe, readily available and promising adult stem cells, which are currently used in several clinical trials. Additionally, their soluble-factor secretion and multi-lineage differentiation capacities place MSCs in the forefront of stem cell types with expected near-future clinical applications. In the present work MSCs were isolated from the umbilical cord matrix (Wharton's jelly) of human umbilical cord samples. The cells were thoroughly characterized and confirmed as *bona-fide* MSCs, presenting *in vitro* low generation time, high proliferative and colony-forming unit-fibroblast (CFU-F) capacity, typical MSC immunophenotype and osteogenic, chondrogenic and adipogenic differentiation capacity. The cells were additionally subjected to an oligodendroglial-oriented step-wise differentiation protocol in order to test their neural- and oligodendroglial-like differentiation capacity. The results confirmed the neural-like plasticity of MSCs, and suggested that the cells presented an oligodendroglial-like phenotype throughout the differentiation protocol, in several aspects sharing characteristics common to those of *bona-fide* oligodendrocyte precursor cells and differentiated oligodendrocytes.

## Introduction

Mesenchymal stem cells (MSCs), also known as mesenchymal stromal cells, are defined as multipotent adult stem cells, possessing self-renewal capacity and multilineage differentiation potential [1], [2]. MSCs were originally identified in the bone marrow [3], but more recently, cells with characteristics similar to MSCs have been identified in many other locations, such as perivascular regions of multiple organs and tissues (like the fat tissue) [4] and several regions of the umbilical cord, namely the umbilical cord matrix (also known as the Wharton's jelly) [5].

MSCs have been characterized as a safe, available, low-immonogenic and clinically promising adult stem cell type [1], [5], [6]. Several reports in the literature have shown the potential of MSCs to differentiate into neural stem-like cells [7]–[9]. Despite controversy about MSCs (a mesenchymal cell type) differentiating into neural-like cellular fates, compelling evidence has shown that indeed MSCs express neuroectodermal markers, like nestin [8], [10]–[13] and have at least a partial neural crest, neuroepithelial origin [14], [15], suggesting plasticity towards neural-like lineages, opening research avenues for the treatment of distinct neurodegenerative diseases [16], [17]. MSCs have been rather explored in terms of neuronal-like differentiation [8], [13], [18]–[20], but the first reports addressing oligodendrocyte-like specification were only published recently [21], [22]. Nevertheless, further studies are required to fully address this potential.

Demyelination of the central nervous system (CNS) is caused by loss of oligodendrocytes (OLs) and may occur as a result of traumatic injury or non-traumatic neurodegenerative diseases, like multiple sclerosis (MS). Remyelination of the affected areas is typically low and demyelinated areas become inflamed and populated by astrocytes, causing the formation of scar tissue [23]. Stem cell-based approaches that allow for a quicker and more robust remyelination of the affected areas are considered promising for the treatment of demyelinating diseases. However, despite recent advances regarding oligodendroglial differentiation of pluripotent stem cells (namely human embryonic stem cells - hESCs [24], [25] and induced pluripotent stem cells - iPSCs [26]), these are not yet considered safe for application in a clinical setting. Hence, the current lack of appropriate and safe cell sources hamper the use of stem cell-based approaches for the treatment of demyelinating diseases in the clinic.

The objectives of the present work were to thoroughly characterize human MSCs isolated from the umbilical cord matrix (UCM) and assess whether these cells possessed neural- and more specifically, oligodendroglial-like differentiation capacity. The results presented here suggest that umbilical cord matrix mesenchymal stem cells (UCM-MSCs) possess a certain degree of plasticity to differentiate into neural-like cells, and subsequently into cells with phenotypic characteristics of oligodendrocyte precursors and immature oligodendrocytes. Despite the need for testing further differentiation protocols and to perform *in vivo* functional studies to assess the full potential of these cells, the results presented here are promising in the context of cell-based therapeutic strategies for demyelinating diseases.

## Materials and Methods

### Isolation and culture of human mesenchymal stem cells (MSCs) from the umbilical cord matrix (UCM)

Human umbilical cords were obtained after birth from healthy donors, with written informed consent of the parent(s) and the study was approved by the Ethics Committee of Maternidade de Bissaya Barreto – Centro Hospitalar de Coimbra (ref. 356/Sec). Samples were stored at room temperature (RT) in sterile 50 ml conical tubes (VWR International) for 12 to 48 h before tissue processing. The isolation procedure of MSCs was adapted from a protocol described by Reinisch *et al.* [27]. Each umbilical cord unit was manipulated under sterile conditions using a class-II biosafety cabinet (Heraeus HS-18) and cut into sections of about 5 cm. The pieces were washed 2 or 3 times using sterile PBS (Sigma-Aldrich), to remove the blood. The umbilical vein was washed with sterile PBS using a 10 ml sterile syringe to remove blood and blood clots. Subsequently, the vein and arteries were removed to avoid endothelial cell contamination. The Wharton's jelly (WJ) was then cut into fragments of 2–5 mm with the help of sterile scalpel and forceps (Fine Science Tools). Groups of approximately 24 fragments were transferred to each 55 cm^2^ tissue culture (TC) plate (Corning) and left to dry for 5 min inside the biosafety cabinet to promote adhesion of the fragments to the polystyrene surface. Once the cord fragments were properly adherent to the plastic, MSC proliferation medium [Alpha-MEM without nucleosides (Life Technologies, cat. no. 22561-021) supplemented with 10% volume/volume (v/v) MSC-qualified Fetal Bovine Serum (FBS) (Hyclone), 10 U/ml of Penicillin, 10 µg/ml Streptomycin and 2.5 µg/ml Amphotericin B (all from Life Technologies)] was added to the culture plate, until all the fragments were completely covered. The fragments were then cultured for 10 days in an incubator (Shel Lab) at 37°C with 5% CO_2_/95% air and 95% humidity, until MSCs started migrating out of the UCM pieces and forming well defined colonies. Then, UCM fragments were removed from the tissue culture plate and cells were passaged.

For the passaging, cells were detached and dissociated using Trypsin (500 µg/ml)-EDTA (200 µg/ml) solution (Life Technologies). Trypsin was inactivated using 10x the volume of MSC proliferation medium, centrifuged (300×*g*, for 5 min at RT), counted and seeded at a density of 3,000 cells/cm^2^ in MSC proliferation medium and maintained in a CO_2_ incubator (as above) until sub-confluence (80–90% confluence).

For cryopreservation, cells were treated in a similar way as described for the passaging procedure, except that cells were resuspended in cryopreservation solution (FBS supplemented with 10% DMSO – Sigma Aldrich) into cryotubes and transferred to a cell freezing container - Mr. Frosty - (both from Thermo Scientific), that was relocated inside a −80°C freezer to achieve a rate of cooling close to 1°C/minute. After 16 h, the cryotubes were transferred to a liquid nitrogen cryotank for long-term storage.

### Proliferation kinetics of UCM-MSCs

MSCs isolated from 3 independent cord samples were continuously cultured from P2 to P8 and counted once they reached 80% confluence at each passage. The population doubling (PD) rate was determined at each passage using the equation *N*_H_/*N*_I_ = 2 *^X^*, or [log_10_(*N*_H_) — log_10_(*N*_I_)]/log_10_(2) = *X*, where *N*_I_ represents the number of cells plated at each passage, *N*_H_ the number of cells harvested at the end of each respective passage and *X* the population doubling (PD), as described [28].

The PD for each passage was calculated and added to the PD of the previous passages to generate data for cumulative population doublings (CPD). In addition, the generation time (GT) - average time between two cell doublings - was calculated from P2 to P8 using the following formula, as described [29]: *X*  =  [log_10_(2) x Δ*t*]/[log_10_(*N*_H_) — log_10_(*N*_I_)].

The total number of cells (TNC) was determined at each passage (P1-P8) by cumulative counting of the cells once they reached a confluence of 80%, using the formula: *X*  =  *N_H_* x B/*N*_I_, in which B represents the total number of cells in the previous passage. TNC accounts for the theoretical number of cells that could be obtained if no cells were discarded between each passage.

### Colony-forming unit-fibroblast (CFU-F) assay

The colony-forming unit-fibroblast (CFU-F) assay of MSCs was determined for 3 independent cord samples at P2 and P8. Cells were seeded at 3 cells/cm^2^ on 55 cm^2^ TC dishes in MSC proliferation medium and cultured for 15 days. One third of the medium was replaced twice a week. Cells were fixed with 4% paraformaldehyde (PFA) (Sigma-Aldrich) for 20 min at RT and stained with Giemsa solution (Sigma-Aldrich). Individual colonies were counted manually.

### Multilineage differentiation of UCM-MSCs

Chondrogenic, adipogenic and osteogenic differentiation capacity of isolated MSCs was assessed using early passage (P2-P3) MSCs. For osteogenic and adipogenic differentiation, cells were plated at 1,000 cells/cm^2^ in the presence of Dulbecco's modified Eagle's medium (DMEM) (Life Technologies) supplemented with 10% MSC-qualified FBS (Hyclone) until reaching 80–100% confluence. Medium was then removed and StemPRO (Life Technologies) osteogenic or adipogenic differentiation medium was added and cells were cultured for 14 days. For chondrogenic differentiation, the cells were plated on low-attachment plates (Corning). A pellet of 5×10^5^ to 1×10^6^ cells was resuspended and droplets of this suspension were plated on the surface of each well. After plating, StemPRO chondrogenic differentiation medium was added and cells were cultured for 14 days.

For osteogenic differentiation assessment, alkaline phosphatase (ALP) and Von Kossa staining were performed. For ALP staining, the cells were washed with PBS and fixed in 10% cold neutral-buffered formalin (Sigma-Aldrich) for 15 min.

Cells were then washed and kept in distilled water for another 15 min and then stained with a 0.1 M Tris-HCl solution (Sigma-Aldrich) containing the substrate Naphtol AS MX-PO4 (0.1 mg/ml) (Sigma-Aldrich) for ALP in dimethylformamide (Fischer Scientific) and 0.6 mg/ml Red Violet LB salt (Sigma-Aldrich) for 45 min. The excess staining was removed by washing 3 times with distilled water. For the Von Kossa staining, the cells were washed with PBS and stained with 2.5% (w/w) silver nitrate (Sigma-Aldrich) for 30 min at RT and then washed 3 times with distilled water.

For adipogenic differentiation assessment, cells were washed with PBS and fixed with PFA using a 2.5% solution for 30 min at RT. Cells were washed once in distilled water and incubated with 0.3% Oil Red-O solution (Sigma-Aldrich) for 1 h at RT, and then washed twice with distilled water.

For chondrogenic differentiation assessment, cells were washed once with PBS and fixed with 2% PFA solution for 30 min at RT. The cells were then washed with PBS and stained with 1% Alcian Blue solution (Sigma-Aldrich) prepared in 0.1 M HCl for 30 min. The excess was removed by washing 3 times with PBS and the cells were then maintained in distilled water.

### Immunophenotypic characterization of UCM-MSCs

The immunophenotypic characterization of UCM-MSCs [30] was performed at passages 2 and 8. Cells were dissociated using StemPro Accutase (Life Technologies), labeled with antibodies against the indicated antigens and analyzed by flow cytometry (FACS Canto II, Becton-Dickinson). The following antibodies were used for the labeling: mouse PO anti-human CD45 IgG1, clone HI30 (5 µL/test) from Life Technologies; mouse APC anti-human CD90 IgG1, clone 5E10 (0.2 mg/ml) from BD Pharmingen; mouse PE anti-human CD105 IgM, clone ALB9 (10 µL/test) from Beckman Coulter; mouse PB anti-human CD11b IgG1, clone ICRF44 (0.2 mg/ml) from BD Pharmingen; mouse Pe-Cy7 anti-human CD13 IgG1, clone WM15 (2.5 µL/test) from BD Pharmingen; mouse PerCP-Cy5.5 anti-human CD34 IgG1, clone 8G12 (5 µL/test) from BD Pharmingen; mouse PE anti-human CD73 IgG1, clone AD2 (10 µL/test) from BD Pharmingen; mouse PE anti-human NFGR IgG1, clone C40-1457 (10 µL/test) from BD Pharmingen.

### Neural-like induction of UCM-MSCs

#### Neuroectodermal-like induction of UCM-MSCs

UCM-MSCs in P3-P7 were dissociated using Trypsin (500 µg/ml)-EDTA (200 µg/ml) solution, counted and seeded on TC dishes at a density of 12,500 cells/cm^2^ in MSC proliferation medium. On the following day, cells were washed with PBS and medium was replaced by neuroectodermal-induction medium [22] composed by DMEM/F12 (Life Technologies) supplemented with N2 (Life Technologies), 10 ng/ml human recombinant epidermal growth factor (EGF) (Peprotech), 10 U/ml of Penicillin, 10 µg/ml Streptomycin and 2.5 µg/ml Amphotericin B. Cells were incubated for 3 days at 37°C, 5% CO_2_/95% air and 95% humidity.

#### Neural stem cell (NSC)-like induction of Neuroectodermal-like induced MSCs (niMSCs)

Neuroectodermal-like induced MSCs (niMSCs) were dissociated using Trypsin (500 µg/ml)-EDTA (200 µg/ml) solution and seeded at a density of 60,000 cells/cm^2^ on non-treated polystyrene (non-tissue culture) aseptic plates (Gosselin) previously treated for 30 min under the UV light of the laminar flow cabinet. Cells were maintained in NSC induction medium [22] [Neurobasal medium (Life Technologies) supplemented with B27 (Life Technologies) and 20 ng/ml of human recombinant EGF and basic fibroblast growth factor (bFGF) (Peprotech), 10 U/ml of Penicillin, 10 µg/ml Streptomycin and 2.5 µg/ml Amphotericin B] in an incubator at 37°C, 5% CO_2_/95% air and 95% humidity for 18 days with medium replacement every 3–4 days.

#### Oligodendrocyte precursor cell (OPC)-like induction of NSC-like cells

NSC-like cells were dissociated into single cells using StemPro Accutase, counted and seeded at 20,000 cells/cm^2^ onto uncoated 6-well TC plates (Corning) or similar plates coated with human purified fibronectin (Roche) or laminin-2/merosin (Millipore). The coatings were performed by covering the well with 10 µg/ml of fibronectin or laminin-2 in PBS at 37°C for 4 h. Plates were then blocked with 0.3% Bovine Serum Albumin (BSA) in PBS for 30 min at 37°C and washed 3 times with PBS before use. The cells were cultured in NSC induction medium [22] with 10 ng/ml of EGF and bFGF. At the end of 3 days, half the medium was replaced by OPC induction medium composed by Neurobasal medium supplemented with B27, 10 ng/ml of human recombinant bFGF, platelet-derived growth factor-AA (PDGF-AA) and 100 ng/ml of Sonic Hedgehog (SHH) (both from Peprotech). At the end of 3 days, the entire medium was replaced by OPC induction medium. Cells were in culture for an additional 12 days period with complete medium changes every 3–4 days.

#### Maturation of OPC-like cells into Oligodendrocyte (OL)-like cells

OPC-like cells were dissociated into single cells using StemPro Accutase, counted and seeded at 20,000 cells/cm^2^ on 96 well TC plates (Corning), coated with 0.1 mg/ml poly-D-lysine (PDL) (Sigma-Aldrich) and 10 µg/ml laminin-2. PDL coating was performed overnight at 37°C by covering the well with 0.1 mg/ml of PDL in PBS and washed 3 times with PBS. Laminin coating was performed by covering the PDL coated well with 10 µg/ml of laminin in PBS and incubated at 37°C for 2 h. The wells were washed 3 times with PBS before use. Cells were cultured in OL differentiation medium, composed by DMEM/F12 supplemented with N2 and 0.5% FBS. T3 (Sigma-Aldrich) and F3/contactin (R&D Systems), were added to the OL differentiation medium in the following conditions: 30 ng/ml of T3 or 10 nM of F3 or both, for a 10 day period. Alternatively, 30 ng/ml of T3 were added in the first 7 days and both factors for the last 3 days of differentiation. The medium was replaced every 3–4 days.

Rat primary astrocytes obtained as described [31], [32] were used as a positive control for astroglial markers assessed by immunocytochemistry analysis. Procedures were performed according to the European Union Directive 86/609/EEC and the legislation Portaria n. 1005/92, issued by the Portuguese Government for the protection of animals used for experimental and other scientific purposes.

### Immunocytochemistry analysis and F-actin staining

Cells were fixed with 4% PFA for 20 min at RT, rinsed with PBS and permeabilized with PBS with 0.1% Triton X-100 for 20 min, except when using anti-human Golgi antibody, where permeabilization was performed using cold acetone for 15 seconds. Cells were incubated in blocking solution (PBS with 0.1% BSA) for 30 min at RT. Cells were incubated with primary antibodies diluted in blocking solution (see below), overnight at 4°C in humidified conditions. The cells were washed with PBS and then incubated with the appropriate fluorescently-labeled secondary antibodies (see below) in PBS with 0.1% BSA for 1 h at RT. Cells were washed and fixed with 4% PFA for 5 min, to crosslink antigen/antibody complexes, washed 3 times and then incubated for 4 min with 200 ng/ml of DAPI (Sigma-Aldrich). To stain cells for polymerized actin (F-actin), a solution of 4 µM FITC-phalloidin (Sigma-Aldrich) in PBS was incubated for 20 min at RT at the end of the immunocytochemistry protocol (after the incubation with the secondary antibodies), and then washed 3 times with PBS to remove unbound reagent.

The following primary antibodies were used: mouse anti-nestin, clone 10C2 (1∶400) from Millipore; mouse anti-O4, clone O4 (1∶400) from R&D Systems; mouse anti-A2B5, clone 105 (1∶500) from R&D Systems; rabbit anti-galactocerebroside (GalC) (1∶50) from Millipore; rabbit anti-MBP (1∶200) from Sigma-Aldrich; rabbit anti-GFAP (1∶500) from Dako Cytomation; rabbit anti-β-III-tubulin IgG1, clone TUJ1 1-15-79 (1∶2000) from Covance and mouse anti-human Golgi, clone 371-4 (1∶30) from Chemicon. The following secondary fluorescently-labeled antibodies were used according to the host species of each primary antibody: Alexa Fluor 488 donkey anti-mouse IgG (H+L) (1∶200) and Alexa Fluor 568 goat anti-rabbit IgG (H+L) (1∶200), both from Life Technologies.

Fluorescence microscopy was performed using a Zeiss Axiovert 200 M microscope using AxioVision Release 4.8 software (Zeiss) for image acquisition. Exposure time was the same for each marker analyzed and for each independent experiment.

### Image analysis and fluorescence quantification

Mean fluorescence intensity (MFI) was quantified using ImageJ software (NIH) for each marker analyzed. In detail, for each marker and for each independent experiment (consisting of cells in all differentiation steps), the intensity of the background and the signal were obtained by determining the background and signal threshold levels (Threshold tool) of at least 3 different fields belonging to the differentiation step with the highest signal intensity. The average threshold levels were then calculated and applied (using the ‘Set threshold’ tool) to all images under analysis (for each marker in each independent experiment). After setting the thresholds, the MFI of the background and the signal were obtained using the Measure tool and the first was subtracted to the latter, analyzing at least 3 fields for each differentiation step. The resulting values were then averaged and subjected to statistical analysis.

### RNA isolation and Real-Time RT-PCR

Total RNA was extracted from cells in culture in different stages of differentiation using the RNeasy Mini Kit (Qiagen) and treated with DNase-I (Qiagen). 250 ng of RNA were used in each duplicate per sample to synthetize cDNA using the SuperScript II Reverse Transcriptase (Life Technologies). Quantitative PCR analysis was done in each duplicate using the total amount of cDNA obtained in each reaction using Power Sybr Green PCR Master Mix (Life Technologies) using a real-time PCR system (7500 Fast real-time PCR System and 7500 Software V2.0.4, Applied Biosystems) and values were normalized to the levels of *actin* and undifferentiated MSCs were used as the control sample. The PCR cycling parameters were 94°C for 5 min; 30 cycles of 30 seconds at 94°C, 1 minute at 60°C, and 1 minute at 72°C; and final extension at 72°C for 10 min. The primers were as follows: *actin* forward, 5′-CAGAAGGATTCCTATGTGGGC-3′, reverse, 5′- GAGGGCATACCCCTCGTAGAT-3′; *fibronectin*, forward, 5′-GAGATCAGTGGGATAAGCAGCA-3′, reverse, 5′- CCTCTTCA-TGACGCTTGTGGA-3′; *sox2*, forward, 5′-CAGGAGAACCCCAAGATGC-3′, reverse, 5′-GCAGCCGCTTAGCCTCG-3′; *nestin*, forward, 5′-CAGCTGGCGCACCTCAAGATG-3′, reverse, 5′- AGGGAAGTTGGGCTCAGGACTGG-3′; *mbp*, forward, 5′-CTGGGCAGC-TGTTAGAGTCC-3′, reverse, 5′-TGGAGCAAAGGTTTGGTGTC-3′; *gfap*, forward, 5′-CTGTTGCCAGAGATGGAGGTT-3′, reverse, 5′-TCATCGCTCAGGAGGTCCTT-3′; *neurofilament*, forward, 5′-GAGCGCAAAGACTACCTGAAGA-3′, reverse, 5′-CAGC-GATTTCTATATCCAGAGCC-3′.

### Co-culture of OPC/OL-like cells with mouse DRG neurons

Purified dorsal root ganglion (DRG) neurons were obtained by a culture system previously described [33] with minor modifications. Briefly, DRGs were dissected from E14-E16 C57BL/6 mice and explants were transferred to 22-mm glass coverslips previously coated with 0.1 mg/ml poly-D-lysine for 1 h at RT followed by 10 µg/ml laminin-1 (Sigma-Aldrich) for 1 h at 37°C. DRG maturation medium was composed by DMEM high glucose supplemented with 10% FBS, 50 ng/ml Nerve Growth Factor (NGF) and 10 U/ml of Penicillin/10 µg/ml Streptomycin (all from Life Technologies). After 2 to 3 days of culture, 0.5 µM Cytosine Arabinose (AraC; Sigma-Aldrich) was added to inhibit proliferating cells. After a 10 day period of DRG maturation, OPC-like cells derived from UCM-MSCs were plated on top of the neurons at a density of 20,000 cells/cm^2^ and the culture remained in OL-like maturation medium supplemented with T3 [as described in the section *Maturation of OPC-like cells into Oligodendrocyte* (*OL*)*-like cells*]. Medium was changed every 3 or 4 days. By the end of 14 days, cells were fixed and immunocytochemistry analysis was performed.

### Statistical analysis

Statistical analysis was performed by Kruskal-Wallis one-way ANOVA followed by Dunn's multiple comparison test or two-tailed Mann-Whitney test (when data was not normal or homoscedastic), or by repeated measures one-way ANOVA followed by Tukey's test (when a parametric analysis of paired data was appropriate), as indicated. Analysis was done using the software GraphPad Prism 5. Values represent mean ± SEM of at least 3 independent experiments (*P<0.05; **P<0.01 and ***P<0.001 for statistically significant differences).

### Protein extraction and quantification, SDS-PAGE, and immunobloting

To obtain protein extracts from MSCs, cells from one TC plate with 21 cm^2^ were detached using StemPro Accutase. The enzyme was inactivated with PBS and then cells were centrifuged (300×*g*, for 5 min at RT). The pellet was washed with PBS and centrifuged again. The supernatant was discarded and the pellet was frozen in liquid nitrogen for 20 seconds and stored at −80°C until further processing. To prepare protein extracts, frozen cell pellets were incubated with 200 µL of cold (4°C) extraction buffer [50 mM Tris-HCl pH 7.4 supplemented with cOmplete, EDTA-free protease inhibitor cocktail (Roche)]. Cells were disrupted using a 200 µl micropipette by repeatedly pipetting up and down on ice and then sonicated on ice, using an ultrasonic cell disrupter (VibraCell - model VCX 750, Sonics & Materials, Inc.) for 1 min (cycles of 1 s pulse interspaced by 1 s) with an amplitude of 40%, to fully disrupt the membrane structure. After centrifugation (at 20,000×*g* at 4°C for 30 min), the supernatants were retrieved and the protein was precipitated by adding 6 volumes of cold acetone (−20°C). Samples were then vortexed and centrifuged (at 20,000×*g* at 4°C for 30 min). The pellets were washed with 90% (v/v) cold acetone and then allowed to dry at RT for 15 min. Pellets were resuspended in 100 µl of 1x sample buffer [58.3 mM Tris/HCl, pH 6.8, 17 µg/ml SDS (BioRad), 50 µl/ml glycerol (GE Healthcare), 15.5 µg/ml DTT (Bioron), 20 µg/ml Bromophenol Blue (GE Healthcare)] and heated at 95°C for 5 min. Protein was quantified using the 2D quant kit (GE Healthcare), according to manufacturer's instructions.

To obtain protein extracts from brain cortex to be used as a positive control for MBP, the brain of one adult female Wistar rat was extracted and stored at −80°C until further processing (procedures were performed according to the European Union Directive 86/609/EEC and the legislation Portaria n. 1005/92, issued by the Portuguese Government for the protection of animals used for experimental and other scientific purposes). To obtain protein extracts, the brain was thawed with cold extraction buffer and then transferred to one centrifuge tube with 750 µL of extraction buffer. The tissue was homogenized using an ultrasonic cell disrupter microtip (VibraCell - model VCX 130, Sonics & Materials, Inc.) with an amplitude of 40–60% (cycles of 1 s pulse interspaced by 1 s) until homogenized. After centrifugation (at 5,000×*g* at 4°C for 5 min), the supernatant was collected. Next, protein was precipitated (200 µL of the total protein extract) and further processes as described to obtain protein extracts from MSCs.

Protein samples (15 µg/lane for rat brain cortex and 35 µg/lane for MSCs extracts) were separated by SDS-PAGE on SDS/discontinuous 4–12.5% (w/v) acrylamide–bisacrylamide (Bio-Rad) gels at constant voltage (80V while samples were in the stacking gel and 120V after entering the resolving gel, for about 1 h 30 min) using a Mini-Protean III (BioRad) apparatus, with running buffer (BioRad).

Blotting was done using polyvinylidene fluoride (PVDF) membranes (BioRad). Membranes were activated by rinsing in methanol, washed with deionized water and then kept in transfer buffer (BioRad). Protein transfer (western-blot) was performed using a Trans-Blot Turbo transfer system (BioRad) according to the manufacturer's instructions, using Trans-Blot Turbo transfer buffer (BioRad).

For immunoblotting, membranes were blocked with blocking solution [PBS-0.1% (v/v) Tween 20 (GE Healthcare) and 5 g/100 ml non-fat dried milk] for 1 h at RT. Incubation with anti-MBP antibody was performed with gentle agitation, overnight at 4°C, followed by 1 h at RT, while staining with anti-GAPDH antibody occurred for 2 h at RT. Membranes were washed with PBS-0.1% (v/v) Tween 20 and then incubated with the respective secondary antibody diluted in blocking solution (1 h at room temperature) and washed with PBS-0.1% (v/v) Tween 20.

Primary antibodies used were diluted in blocking solution and were rabbit monoclonal (clone EP1448Y) anti-MBP (Abcam, Ab53294) diluted 1∶1,000 and mouse monoclonal anti-GAPDH (Santa Cruz Biotechnology, SC-365062) diluted 1∶500. The respective secondary antibodies were also diluted in blocking solution and were goat anti-rabbit antibody conjugated with alkaline phosphatase (Jackson ImmunoResearch Laboratories, Inc.) diluted 1∶6,000 and donkey anti-mouse antibody conjugated with Alexa 488 (Life Technologies) diluted 1∶500.

An ECF (enhanced chemiflorescence) kit (GE Healthcare) was used for detection of the alkaline phosphatase-conjugated antibody, according to the manufacturer's instructions. Alkaline phosphatase activity (using ECF) and Alexa 488 fluorescence was visualized on a Molecular Imager FX system (BioRad) using the software Quantity One (BioRad). The integrated density of the western-blot bands was obtained using Image J software, using the ‘Threshold’ tool, followed by the command ‘Analyze Particles’.

## Results

### Isolation, expansion and characterization of human umbilical cord matrix mesenchymal stem cells (UCM-MSCs)

Human MSCs were isolated from 12 umbilical cords, as described in the ‘Materials and Methods’ section, with a success rate of 100% (in agreement to what has been previously reported [34]). At the end of 5 to 10 days in culture, several Wharton's jelly fragments were still attached to the tissue culture (TC) dishes and showed cells migrating from the tissue. Colonies of cells displaying an MSC-like phenotype, with spindle-shaped morphology could be readily identified by phase-contrast microscopy (Figure S1). Cells proliferated rapidly and formed compact colonies by the end of 10-14 days *in vitro*. Then, the cells were detached and dissociated and by the end of Passage 1 (P1) constituted a homogeneous monolayer with MSC-like morphology (adherent, spindle-shaped fibroblastoid-like cells). The cells were then either cryopreserved or expanded until passage 8 (P8) and further characterized.

**Figure 1:**
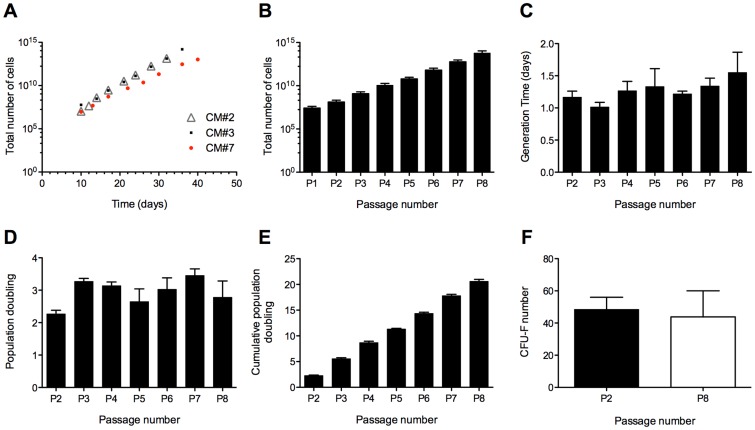
Proliferation and colony-forming capacity of human umbilical cord mesenchymal stem cells (hUCM-MSCs) *in vitro*. Total number of cells (hUCM-MSCs) along time (days) and passage number (*A, B*), generation time (*C*), population doubling (*D*), cumulative population doubling (*E*) from passage 1 (P1) or P2 until P8, as indicated, and colony forming unit-fibroblast (CFU-F) number at P2 and P8 (*F*). Cells were isolated from 3 distinct human umbilical cord matrix samples (CM#2, #3 and #7). From P2 onwards (inclusively), cells were plated at a fixed density of 3,000 cells/cm^2^, allowed to proliferate until sub-confluence and re-plated in the same way (*A-E*), or plated at 3 cells/cm^2^ at P2 and P8 and cultured for 15 days for the CFU-F study (*F*). The total number of cells (TNC) was determined at each passage (P1-P8) by cumulative counting of the cells once they reached a confluence of 80% (*A, B*). The TNC designates the theoretical number of cells that could be obtained if no cells were discarded between each passage. The observed mean generation time (GT) was between 1.02 (±0.071) and 1.55 (±0.316) days (*C*), and no statistically significant differences were found in GT from passages 2 to 8 (Kruskal-Wallis one-way ANOVA followed by Dunn's Multiple Comparison test). The CFU-F capacity was maintained from P2 to P8 (*F*) and no statistically significant differences were found (two-tailed Mann-Whitney test). Bars represent mean ± SEM (*B-F*).

UCM-MSCs have been described to proliferate readily *in vitro* [34], [35], hence we sought to thoroughly characterize their proliferation kinetics, namely the total number of cells - TNC (Figures 1A, B), generation time - GT (Figure 1C), population doubling - PD (Figure 1D) and cumulative population doubling - CPD (Figure 1E) of cells isolated from 3 randomly selected independent samples (CM#2, CM#3 and CM#7) between passages 2 and 8 (P2-P8). Notably, by the end of P4 (17 to 21 days after the initial isolation of the UCM explants), the total number of cells obtained from each sample (i.e., if no cells had been discarded until that point) had surpassed 1×10^9^ cells (Figures 1A, B), well above what is considered relevant for clinical applications [29] (about 1–2 million cells per kg of body weight) and at passage 8 the TNC was 5.98×10^13^ (±4.76×10^13^). On average, between P2 and P8, the generation time ranged from 1.02±0.071 (SEM) to 1.55±0.316 (SEM) days (Figure 1C). Moreover, the colony-forming unit-fibroblast (CFU-F) capacity of the cells (Figure 1F) was maintained throughout P2 to P8 [48.5±7.52 (SEM) and 43.8±16.20 (SEM)]. For both types of assays (GT and CFU-F), no statistically significant differences were found between the time points, indicating that neither the generation time nor the CFU-F capacity were significantly affected by passaging the cells until P8. Hence, the cells showed high proliferative capacity typical of *bona-fide* MSCs, maintained a short generation time from passages 2 to 8 and reached a clinically relevant number (superior to 1×10^9^ cells) within a time-frame of 17 to 21 days.

### Multilineage differentiation capacity and immunophenotypic characterization of UCM-MSCs

**Figure 2:**
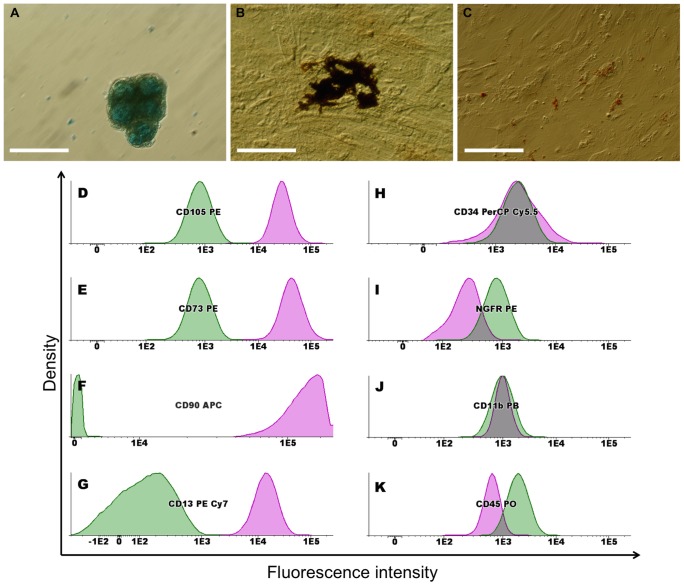
Multilineage differentiation potential and immunophenotypic characterization of hUCM-MSCs. The multilineage differentiation potential of hUCM-MSCs was demonstrated after 14 days in culture, under conditions that favour chondrogenic, osteogenic or adipogenic differentiation. Phase contrast images of cells stained with Alcian Blue for chondrogenesis (*A*), ALP and von Kossa for osteogenesis (*B*) or Oil Red-O for adipogenesis (*C*). Scale bar represents 100 µm. For immunophenotypic characterization, hUCM-MSCs at passage 2 were dissociated using Accutase (Life Technologies), labelled with antibodies against the indicated antigens and analysed by flow cytometry. Cells were positive for CD105 (*D*), CD73 (*E*), CD90 (*F*) and CD13 (*G*) and negative for CD34 (*H*), NGFR (*I*), CD11b (*J*) and CD45 (*K*) (pink lines) when compared with unlabelled MSCs (green lines), as depicted in the histograms. Histograms were obtained from one sample (UCM#2) at passage 2 and are representative of 3 independent samples at P2 and P8.

MSCs are multipotent stem cells that can differentiate *in vitro* into chondrocytes, osteocytes and adipocytes [2]. The multilineage differentiation potential of UCM-MSCs was demonstrated in culture (Figure 2A-C), under conditions that favor osteogenic, adipogenic or chondrogenic differentiation (Materials and Methods section). Chondrogenic induction was observed by Alcian Blue staining (Figure 2A), while osteogenic differentiation was evident by an increase in alkaline phosphatase (ALP) activity (reddish areas) and enhanced mineralization showed by von Kossa staining (dark areas), as shown in Figure 2B. Adipogenic induction, although less efficient, was visible by the cellular accumulation of lipid-rich vacuoles that stained with Oil Red-O (Figure 2C). These results indicate that UCM-MSCs possess the multilineage differentiation capacity characteristic of MSCs [2].

Flow cytometry analysis showed that UCM-MSCs were positive for CD13, CD73, CD90 and CD105, while cells did not express CD34 and CD45 (hematopoietic lineage markers), CD11b and NGFR (Figure 2D-K and Table 1 represent the analysis of one sample in passage 2, while data from 3 independent samples at passage 2 and 8 is presented in Figure S2), showing that this phenotypic profile was consistent between different donors and with the MSC phenotype previously described by us [36] and others [30], [37], [38].

**Table 1:** Summary of the flow cytometry analysis of UCM-MSCs.

Positive markers	Negative markers
CD13	CD11b
CD73	CD34
CD90	CD45
CD105	NGFR

### Differentiation of UCM-MSCs into an oligodendrocyte-like lineage

**Figure 3:**
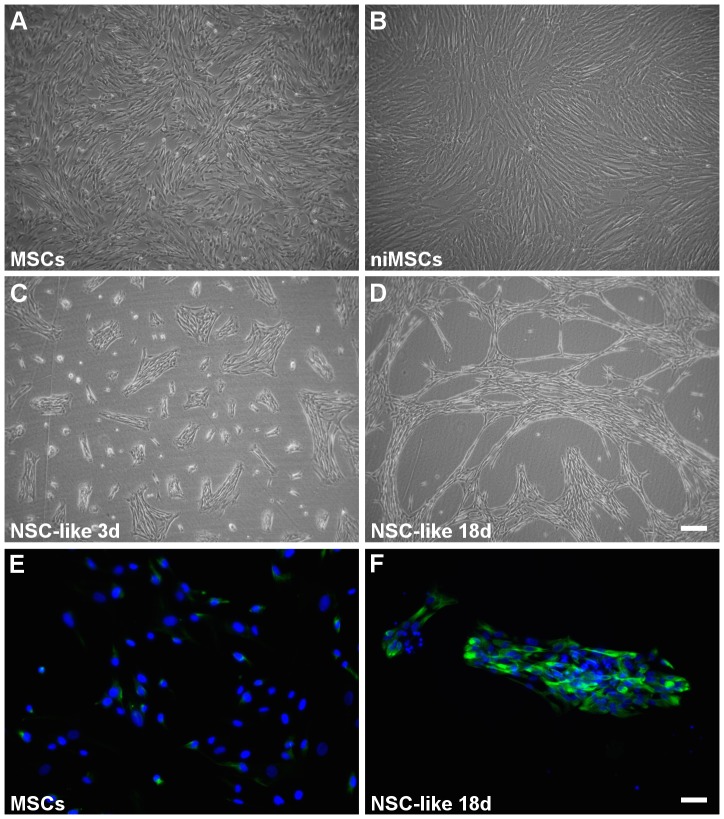
Cell morphology and expression of nestin in hUCM-MSC and during the first steps of differentiation. Phase contrast images of undifferentiated hUCM-MSCs (*A*), neuroectodermal-like induced MSCs – niMSCs (*B*) and NSC-like cells after 3 (*C*) and 18 days (*D*) in culture in NSC induction medium. Immunofluorescence microscopy images for the neural precursor marker nestin (in green) in undifferentiated MSCs (*E*) and NSC-like cells (*F*). Counterstaining of nuclei was performed with DAPI (in blue). In (*A-D*) the scale bar represents 200 µm and in (*E-F*) represents 50 µm. Images are representative of at least 3 independent experiments.

**Figure 4:**
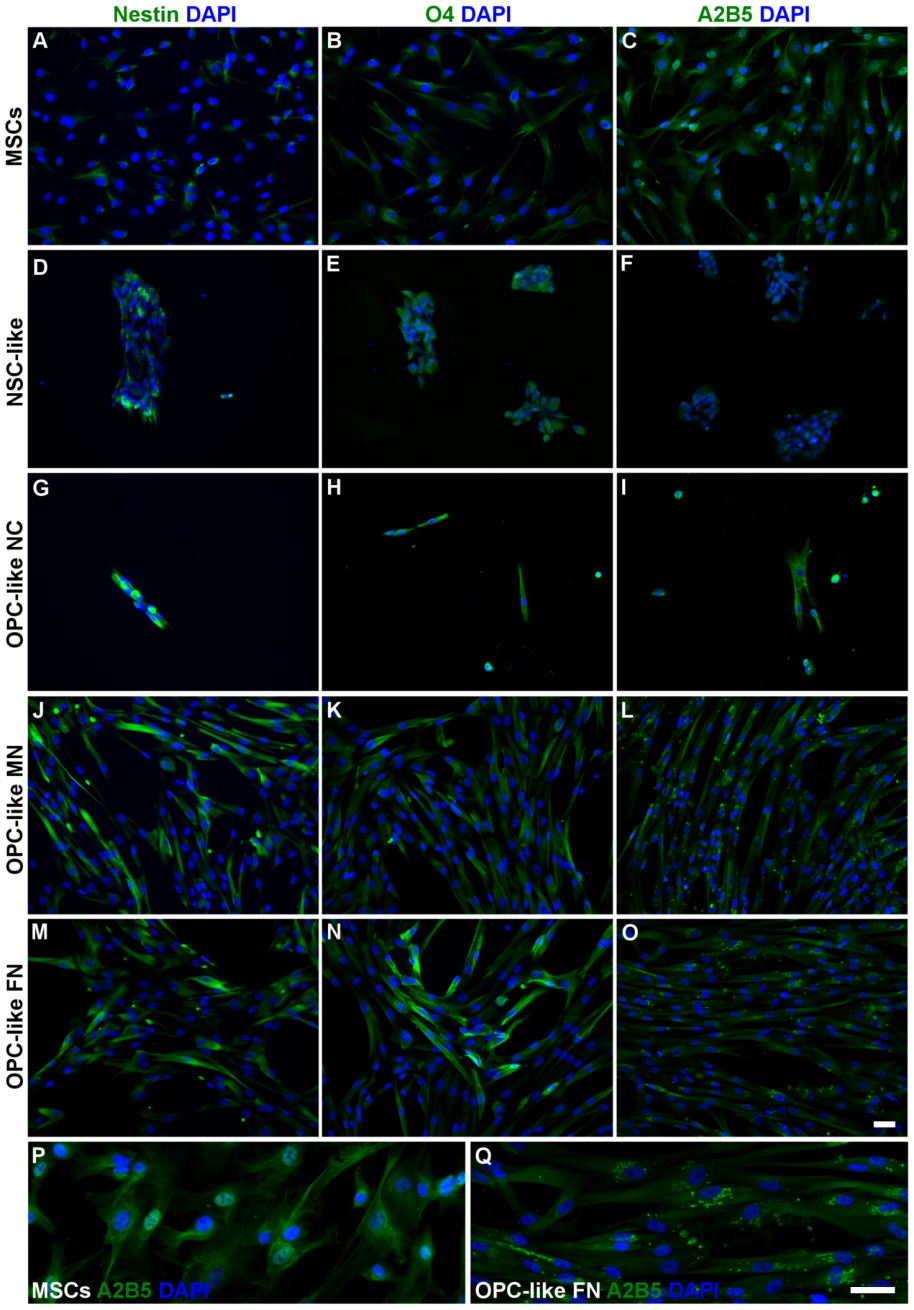
Expression of neural- and oligodendroglial-precursor markers in hUCM-MSCs, NSC- and OPC-like cells. The expression of nestin (neural precursor marker), A2B5 (OPC marker) and O4 (OPC/OL marker) in undifferentiated hUCM-MSCs (*A-C*), after neural-like induction (*D-F*) and OPC-like induction using non-coated (*G-I*), laminin-2 (merosin-MN)-coated (*J-L*) and fibronectin (FN)-coated (*M-O*) tissue culture polystyrene was determined by immunofluorescence microscopy (in green). (*P*) and (*Q*) are inserts of (*C*) and (*O*) images, respectively, to highlight the presence of the typical OPC punctate and perinuclear distribution of A2B5 in OPC-like cells (*Q*), in contrast with control hUCM-MSCs (*P*). Counterstaining of nuclei was performed with DAPI (in blue) and the scale bars represent 50 µm. Images are representative of at least 3 independent experiments.

**Figure 5:**
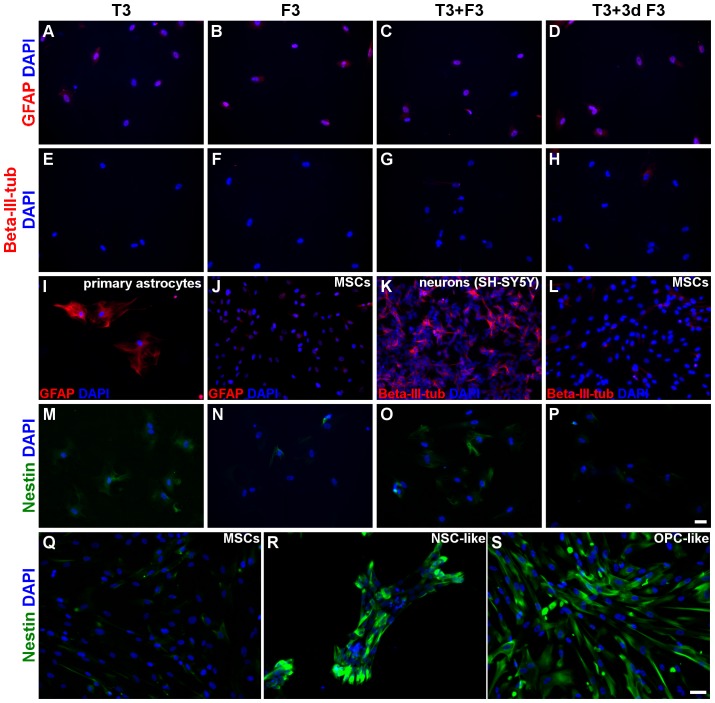
Expression of neural precursor, astrocytic and neuronal markers by OL-like cells. The expression of the astrocytic marker GFAP (*A-D and I-J*), the neuronal marker beta-III-tubulin (*E-H and K-L*) and the neural precursor marker nestin (*M-P and Q-S*) in OL-like cells derived from OPC-like cells cultured on fibronectin (*A-H and M-P*) was determined by immunofluorescence microscopy. OL-like cells were differentiated for 10 days on laminin-2 (merosin)-coated wells, in the presence of T3 (thyroid hormone), F3 (contactin), T3+F3 or 7 days T3 followed by 3 days T3+F3 (T3+3d F3), as indicated. It was evident that the neural precursor marker nestin decreased in the OL-like stage of differentiation (*M-P*), as compared to NSC- or OPC-like cells (*R and S, respectively*), indicating maturation of the OL-like cells. It was also clear that the astrocytic marker GFAP was present in rat astrocytes as expected (*I*), but essentially absent in OL-like cells (*A-D*) or hUCM-MSCs (*J*). Similarly, the neuronal marker beta-III-tubulin was detected in the neuronal cell line SH-SY5Y (*K*) but undetected in OL-like cells (*E-H*) or hUCM-MSCs (*L*). Counterstaining of nuclei was performed with DAPI (in blue) and the scale bars represent 50 µm - scale bar in image (*P*) is representative for images (*A-P*) and scale bar in image (*S*) is representative for images (*Q-S*). Images are representative of at least 3 independent experiments.

**Figure 6:**
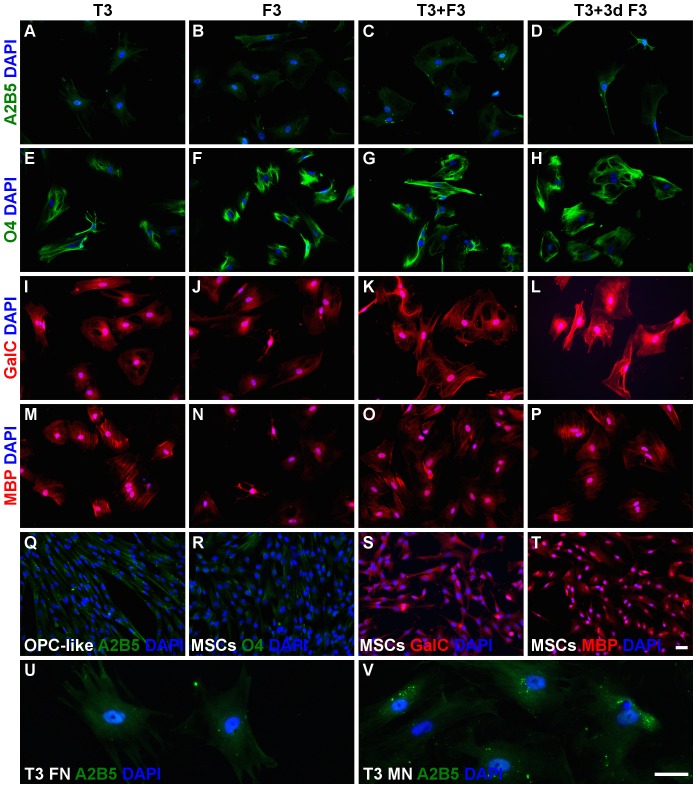
Expression of oligodendroglial markers by OL-like cells. The expression of the OPC marker A2B5 (*A-D*), the OPC/OL marker O4 (*E-H*) and the OL markers GalC (*I-L*) and MBP (*M-P*) in OL-like cells derived from OPC-like cells cultured on fibronectin was determined by immunofluorescence microscopy. OL-like cells were differentiated for 10 days on laminin 2 (merosin)-coated wells, in the presence of T3 (thyroid hormone), F3 (contactin), T3+F3 or 7 days T3 followed by 3 days T3+F3 (T3+3d F3), as indicated. It could be observed that the OPC marker A2B5 greatly decreased in the last stage of differentiation (*A-D*), as compared to OPC-like cells (*Q*), indicating maturation of the OL-like cells. The decrease was also more evident when OL-like cells were derived from OPC-like cells cultured on fibronectin (FN)-coated wells (*U*) than in laminin-2/merosin (LM/MN)-coated wells (*V*). The OPC/OL marker O4 was expressed at high levels in OL-like cells (*E-H*), in contrast to the low levels in hUCM-MSCs (*R*). The OL marker GalC was more highly expressed in OL-like cells (*I-L*) as compared to MSCs (*S*), while MBP seemed to be expressed (*M-P*) at similar levels (*T*). Counterstaining of nuclei was performed with DAPI (in blue) and the scale bars represent 50 µm - scale bar in image (*T*) is representative for images (*A-T*) and scale bar in image (*V*) is representative for images (*U-V*). Images are representative of at least 3 independent experiments.

After assuring the fidelity of the UCM-MSCs obtained, we focused on the differentiation of these cells into oligodendrocyte-like cells using a stepwise protocol, based on the literature for neural- and oligodendroglial-like induction of distinct types of stem cells, including embryonic stem cells [24], [25], [39] and MSCs [7], [9], [22]. MSCs were subjected to distinct soluble factors, culture surfaces and coating conditions during the differentiation process (Figure S3). Briefly, undifferentiated MSCs (Figures 3A and 4A-C) were treated with epidermal growth factor (EGF) for 3 days for generation of neuroectodermal-induced MSCs (niMSCs) (Figure 3B). Next, niMSCs were cultured on non-treated polystyrene in the presence of EGF and basic fibroblast growth factor (bFGF) for induction into neural stem-like (NSC-like) cells (Figures 3C, D, F and 4D-F). The latter were then cultured on tissue culture polystyrene using different coating conditions (Figure S3) for the generation of oligodendrocyte precursor-like (OPC-like) cells (Figure 4G-O). Finally, these progenitor cells were seeded on poly-D-lysine (PDL) and laminin-2/merosin (MN) double-coated surfaces for the generation of oligodendrocyte-like (OL-like) cells (Figures 5 and 6).

### Induction of MSCs into neural-like precursor cells

MSCs used for differentiation experiments were taken from cryopreserved cell batches between passages 3 and 7 obtained from 3 independent donors, characterized as described above (Figures 1 and 2). After 3 days under neuroectodermal-inducing conditions (in the presence of N2 and EGF) the niMSCs kept the spindle-shape morphology (Figure 3B) characteristic of MSCs (Figure 3A) and seemed to maintain proliferation.

**Figure 7:**
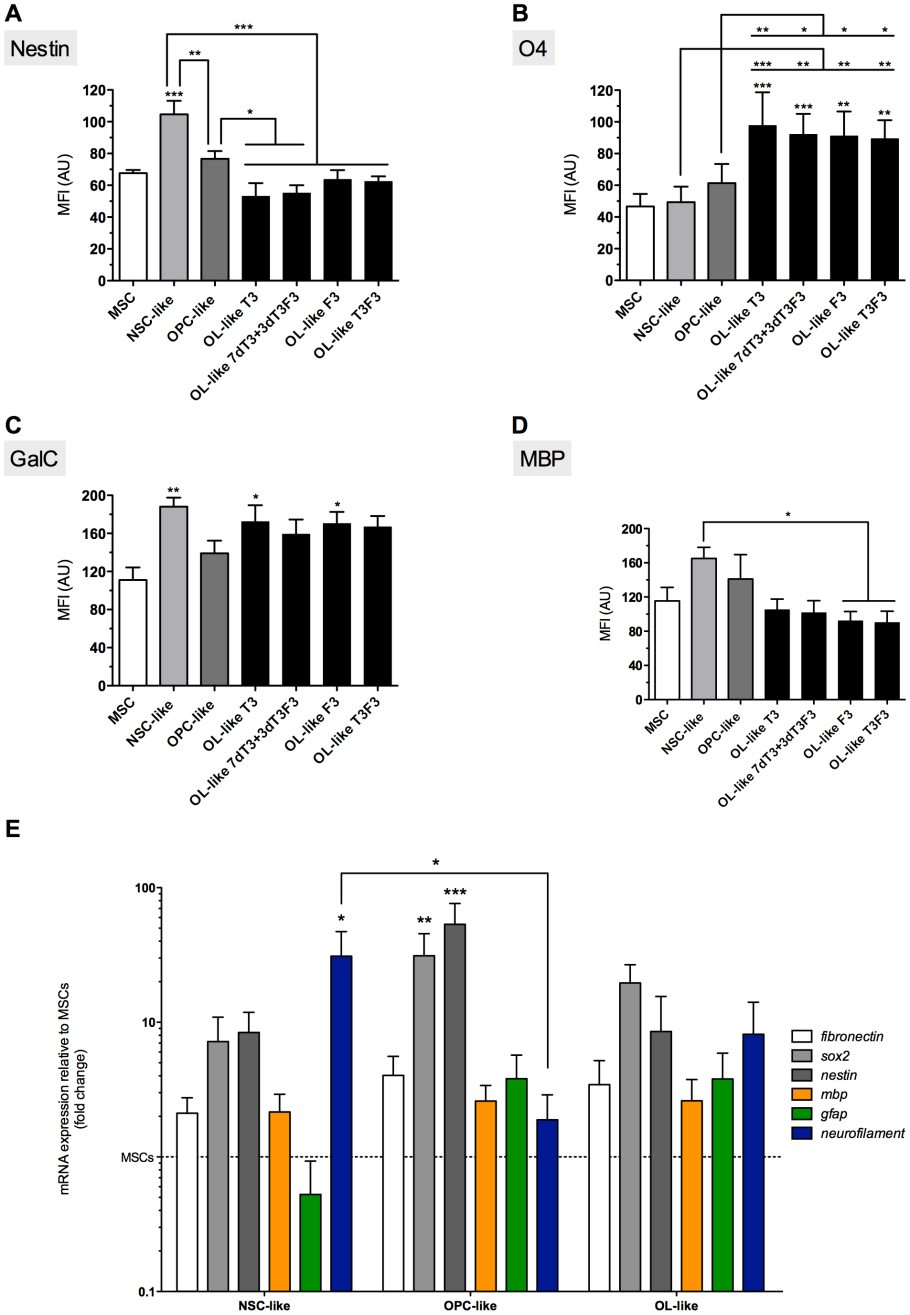
Quantification of mean fluorescence intensity (MFI) of immunofluorescence images and mRNA expression of differentiation stage markers. MFI values for nestin (*A*), O4 (*B*), GalC (*C*) and MBP (*D*) of immnunofluorescence images (representative images on Figures 4 to 6) were quantified using Image J software. The graphics represent results regarding cells cultured during the OPC-like differentiation stage on fibronectin-coated TC polystyrene. OL-like cells were differentiated for 10 days on laminin 2 (merosin)-coated wells, cultured in the presence of T3, F3, T3F3 or 7dT3+3dT3F3, as indicated. Bars represent mean ± SEM of at least 3 independent experiments. Statistical analysis was performed by repeated measures one-way ANOVA followed by Tukey's Multiple Comparison Test. Statistically significant differences for each marker between MSCs and cells at other differentiation stages were indicated on top of the corresponding graphic bars, while differences between other conditions were represented using connectors (*P<0,05; **P<0,01 and ***P<0,001). Real time RT-PCR analysis (*E*) was performed for genes representative of neural progenitors (*sox2* and *nestin*), neurons (*neurofilament*), astrocytes (*gfap*) and oligodendrocytes (*mbp*), while *fibronectin* was used as a mesenchymal marker. Bars represent mean ± SEM of at least 3 independent experiments and are expressed as fold change of 2^−ΔΔCt^ using *actin* as a reference gene and undifferentiated MSCs as the control condition. Statistical analysis was performed by Kruskal-Wallis one-way ANOVA followed by Dunn's multiple comparison test (*P<0,05, **P<0,01 and ***P<0,001).

In order to induce the differentiation of niMSCs into a NSC-like phenotype, cells were seeded on non-treated polystyrene plates (sterile, non-tissue culture ‘bacterial dishes’) and cultured up to 18 days (Figure 3C,D). The induction medium contained the soluble factors bFGF and EGF, described to favor neuroectodermal-like fate of MSCs [7], [9]. After 3 days in culture (Figure 3C), cells had formed patches or small colonies that were maintained until 18 days in NSC-induction medium (Figure 3D). Although undifferentiated MSCs already expressed nestin [11] to a certain extent (Figures 3E and 4A), there was an increase in the expression of this NSC marker after the NSC-like induction phase, as addressed by immunocytochemistry (Figures 3F and 4D). The quantification of the mean fluorescence intensity (MFI) of immunofluorescence images (see Materials and Methods) was addressed for distinct neural markers at the different stages of differentiation under study (Figure 7A-D). The MFI of nestin in cells after NSC-like induction (NSC-like) showed a significant increase compared to undifferentiated MSCs (Figure 7A).

No substantial expression of the oligodendrocyte precursor markers A2B5 or O4 (the latter being also present in mature oligodendrocytes) could be detected at the NSC-like stage or in undifferentiated MSCs (Figures 4 E/F and B/C, respectively), and the faint staining visible in the immunocytochemistry images seem to represent background staining, as will become evident when comparing MSCs with cells at more mature differentiation stages (Figures 4, 6 and 7B). Interestingly, galactocerebroside (GalC, a marker of mature oligodendrocytes) seems to be already expressed by undifferentiated MSCs (Figure 6S), confirming observations made by others [7]. However, its expression was significantly increased after NSC-like induction (Figure 7C). The expression of myelin basic protein (MBP), a protein expressed by mature oligodendrocytes, also showed a tendency to increase during the NSC-like differentiation stage (Figure 7D). Nevertheless, MBP could already be detected in low amounts in undifferentiated MSCs, as determined by immunocytochemistry (Figures 6T and 7D) and semi-quantitative RT-PCR (not shown), in agreement with results by others [7].

### Oligodendrocyte precursor cell (OPC)-like induction

At the end of the NSC-like induction protocol, cells were detached and seeded on 6-well plates coated with fibronectin (FN), described to play an essential role in survival and proliferation signaling during the OPC differentiation stage [40]. In parallel, cells were also seeded on laminin-2/merosin (MN) coated wells, as previously described by Zhang and colleagues [22] for the differentiation of MSC into OPC-like cells. As control, cells were also plated on non-coated (NC) tissue culture wells.

By the end of the OPC-like induction stage, the cells cultured on FN- or MN-coated plates survived well and maintained the spindle shape morphology, when compared with cells cultured on uncoated wells, which did not attach well to the surface and only some small adherent clusters and single cells survived until the end of this stage (Figure 4G-O).

Figure 4 shows that there seemed to be a tendency for increased expression of Nestin, O4 and A2B5 in OPC-like cells (regardless of the coating conditions used) when compared with undifferentiated MSCs. The quantification of the MFI of Nestin and O4 (Figure 7A,B) showed that despite not being statistically significant, there was a slight increase of these markers in OPC-like cells cultured in FN when compared with MSCs. RT-PCR analysis showed that nestin was in fact upregulated in terms of gene expression in OPC-like cells when compared to undifferentiated MSC (Figure 7E). The presence of Nestin in OPC-like cells is in agreement with the literature, since it has been reported that OPCs may express nestin [41], [42]. Nonetheless, there seems to be a peak of Nestin expression during the NSC-like stage, and then a significant decrease at the OPC-like stage (Figure 7A), which is consistent with a NSC-like stage followed by an OPC-like stage of differentiation. Although MFI analysis of the levels of O4 between OPC-like cells and undifferentiated MSCs showed that these differences were not statistically significant (Figure 7B), there was a trend of increased expression of O4 on OPC-like cells when comparing with MSCs (Figure 4B and N), indicating at least some degree of oligodendroglial-like commitment of the cells. Moreover, the presence of A2B5 was visible in OPC-like cells (especially when cultured on FN or MN-coated surfaces) when compared to MSCs (Figure 4C, I, L, O, P and Q). Although it was not possible to quantify the MFI of this marker (due to its punctate distribution), it can be readily perceived in Figures 4P and Q (which show a higher magnification of the images depicted in Figures 4C and O, respectively) that the punctate and perinuclear distribution of A2B5 similar to that reported in *bona-fide* OPCs [42] was only visible in OPC-like cells and not in MSCs.

Overall, the protocol tested using FN or MN-coated tissue culture polystyrene (TCPS) seemed to favor a neural- and oligodendroglial precursor-like fate of MSCs.

### Oligodendrocyte (OL)-like induction

OPC-like cells were detached and dissociated from the non-coated, fibronectin- or laminin-coated TCPS and re-seeded on PDL and MN double-coated 96 well plates, in distinct OL differentiation media (see Materials and Methods). The presence of merosin is known to favor oligodendrocyte differentiation [43], hence this extracellular matrix (ECM) protein was chosen as a coating element for the final stage of the protocol.

In order to induce the differentiation of OPC-like cells into OL-like cells, the thyroid hormone T3 (known to induce the differentiation of oligodendrocyte progenitor into mature OLs [44]), F3/Contactin (a ligand known to induce the maturation of oligodendrocytes [21], [22], [45]), or T3 combined with F3 (T3F3) were added to the basal differentiation media and incubated for 10 days. It was also tested the effect of T3 in the first 7 days of culture and T3 together with F3 during the last 3 days of differentiation (7dT3+3dT3F3).

Immunocytochemistry analysis of nestin in OL-like cells previously cultured on FN-coated TCPS during the OPC-like stage (the preceding differentiation step) showed that the expression of this neural stem/progenitor marker decreased significantly during the final stage of differentiation in all the conditions tested in the final differentiation stage when compared with NSC-like cells, or when compared with OPC-like cells when differentiated terminally in the presence of 7dT3+3dT3F3 or T3 (Figures 5M-S and 7A). Nestin levels peaked at the NSC-like stage and then decrease during the final differentiation step (OL-like stage), to levels similar to those of undifferentiated MSCs (Figures 5M-S and 7A). These results support the idea that the cells underwent a neural progenitor-like state during the NSC- and OPC-like stages of the differentiation protocol and then differentiated into a more mature phenotype at the OL-like stage (similar to what has been described for *bona-fide* OLs [46]).

Reinforcing this idea was the behavior of the OPC marker A2B5 [46], which decreased during the final stage of differentiation (OL-like cells) when compared with OPC-like cells (Figure 6 A-D, Q, U, V). This effect was more evident in oligodendrocyte-like cells that had been previously cultured on FN-coated dishes during the OPC-like differentiation stage than in cells that had previously been cultured on MN-coated TCPS (Figure 6U, V). The loss of this OPC marker indicates that OL-like cells derived from OPC-like cells cultured on FN-coated wells might be more mature than those obtained from OPC-like cells cultured on MN-coated or non-coated wells, in agreement with reports highlighting the important role of fibronectin during the OPC stage of differentiation [40].

To further characterize the OL-like cells obtained, we screened for the presence of neural maturation markers for astrocytic (GFAP), neuronal (beta-III-tubulin) and oligodendroglial (O4, GalC and MBP) lineages. The images of OL-like cells presented in Figures 5 and 6 were obtained from cells that had previously been cultured on FN-coated TCPS during the OPC-like state, and from this point onwards will be considered as the main condition under analysis during the final stage of differentiation, unless stated otherwise.

The expression of GFAP was negative in OL-like cells in all differentiation conditions (Figure 5 A-D), similar to what had been observed in undifferentiated MSCs (Figure 5J) and in contrast to what was shown in Figure 5I, where primary rat astrocytes were used as positive control for this marker. Cells that were cultured on laminin-2 or non-coated wells during the OPC-like stage showed some expression of this astrocytic marker in the final differentiation stage (OL-like stage) - data not shown. These results further supported the idea that the oligodendroglial-like fate of MSCs seems to be favored by the presence of FN during the OPC-like stage.

In Figure 5E-H, it can be observed that the expression of beta-III-tubulin was essentially absent in OL-like cells obtained in the presence of any of the distinct differentiation media, although there was some expression in cells that were cultured on non-coated wells during the OPC-like stage (data not shown). In Figure 5K, the SH-SY5Y cell line was used as a positive control for this neuronal marker, which was absent in undifferentiated MSCs (Figure 5L).

The expression of O4, an OPC marker which is maintained in mature oligodendrocytes [46], increased during the differentiation of MSCs into OL-like cells (Figure 6E-H). For cells cultured on fibronectin during the OPC-like stage, the quantification of the MFI of O4 showed a significant increase between MSCs, NSC-like or OPC-like cells and the cells obtained during the final stage of differentiation (OL-like cells) cultured in the presence of any combination of differentiation factors (Figure 7B). Similar results were obtained for cells previously cultured on non-coated TCPS, or for cells maintained on MN-coated TCPS during the OPC-like stage when finally differentiated in presence of F3 only (data not shown).

GalC seemed to be already expressed at some extent by undifferentiated MSCs (Figure 6S). However, the expression of this oligodendrocyte marker increased in oligodendrocyte-like cells differentiated under all tested conditions (Figure 6I-L). The MFI of GalC showed a tendency to increase between MSCs and OL-like cells in all conditions. Compared to MSCs, the level of GalC expressed by OL-like cells was significantly increased only in OL-like cells cultured in the presence of T3 or F3 (Figure 7C). Unexpectedly, the presence of GalC was also higher in NSC-like cells, when compared with undifferentiated MSCs. Nevertheless, only during the final stage of differentiation (in OL-like cells) it was possible to observe cells with a branched morphology similar to oligodendrocytes and strongly positive for GalC, like those terminally differentiated in the presence of T3 or F3 (asterisk in Figures 6I and J, respectively). For cells cultured on non-coated wells during the OPC-like stage, there was a significant increase in the expression of GalC between OL-like cells (cultured in the presence of any of the final differentiation factors) and MSCs, but no significant differences were found between OL-like cells derived from OPC-like cells cultured on laminin-coated wells and MSCs (data not shown).

Immunocytochemistry analysis of MBP showed that this mature oligodendrocyte marker was present in OL-like cells (Figure 6M-P) after the final differentiation step, however, the levels of MBP were similar to those observed in undifferentiated MSCs, as illustrated in Figure 6T (the presence of MBP in MSCs was confirmed by western-blot analysis – Figure S4). The quantification of the MFI throughout the distinct stages of the differentiation protocol showed that the expression of MBP had a tendency to increase (although the differences were not statistically significant) during the NSC- and OPC-like stages of differentiation (Figure 7D). In fact, there was a significant decrease in the expression of MBP in the final step of differentiation, between NSC-like and OL-like cells cultured in the presence of F3 or T3F3, but not T3 or 7dT3+3dT3F3 (Figure 7D). Also, for cells cultured on uncoated surfaces during the OPC-like stage, there was a significant decrease in the expression of MBP between NSC-like cells and oligodendrocyte-like cells cultured in the presence of F3 and between OPC-like cells and OL-like cells cultured in the presence of T3 or F3 (not shown).

The expression of the oligodendrocyte markers MBP, GalC and O4, together with the downregulation of nestin and A2B5 in the OL-like differentiated cells, supports the idea that MSCs could be induced to differentiate into OL-like cells, sharing the behavior of several markers expressed by *bona-fide* oligodendrocytes and its precursors [46].

The more evident downregulation of A2B5 and the absence of neuronal (beta-III-tubulin) and astrocytic (GFAP) markers observed in OL-like cells derived from OPC-like cells cultured on fibronectin, suggests that these coating conditions (during the OPC-like stage) are suitable for the differentiation of MSCs into oligodendrocyte-like cells. Hence, UCM-MSCs could be differentiated into NSC-like cells, OPC-like cells and finally into oligodendrocyte-like cells, with expression of specific markers for each stage of differentiation. Figure 6 I and J (staining for GalC) illustrates terminally differentiated OL-like cells displaying a branched morphology (asterisks within images), resembling oligodendrocytes. Despite several cells with this morphology could be observed after the final differentiation step, they represented a minority of the cells.

### Pattern of expression of MBP

**Figure 8:**
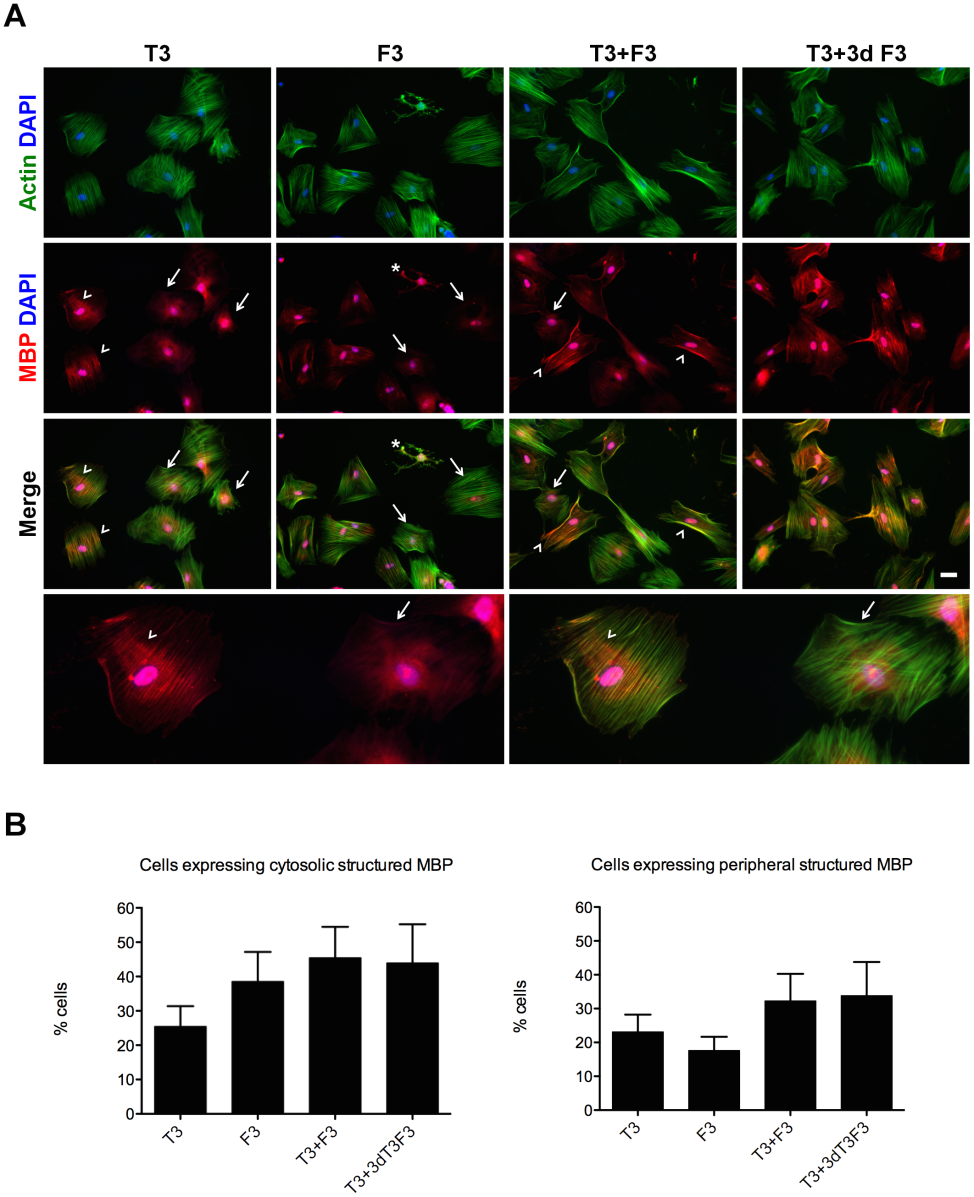
Co-localization of MBP and F-actin in oligodendrocyte-like cells. Fluorescence microscopy images of OL-like cells (*A*) stained with fluorescent phalloidin (in green) to assess the presence of polymerized actin (F-actin) and an antibody against MBP (in red) and both channels merged, as indicated. Cells were derived from OPC-like cells cultured on FN-coated wells. Cells showing structured MBP and with partial co-localization with F-actin (arrow heads) or with diffuse MBP and no co-localization with F-actin (arrows) were visible after OL-like differentiation in the presence of T3, F3, T3+F3 or 7 days T3 followed by 3 days T3+F3 (T3+3d F3), as indicated. The bottom panel is a magnification of the T3 condition. Images are representative of at least 3 independent experiments and scale bar corresponds to 50 µm. The percentage of cells expressing structured MBP was calculated (*B*) under two categories: cells expressing structured cytoplasmic (left) or peripheral (right) MBP. Values were expressed as percentage of total cells and bars represent mean ± SEM percentage of cells present in at least 14 fields, belonging to 3 independent experiments. No statistically significant differences were found between the distinct differentiation conditions (Kruskal-Wallis one-way ANOVA followed by Dunn's multiple comparison test).

Although we could not observe an increase of MBP expression in OL-like cells when compared with undifferentiated MSCs, it could be observed that there were distinct patterns of expression of MBP in the differentiated cells. One of the patterns was common to all stages of differentiation, including undifferentiated MSCs (Figure 6T), which was a strong staining in the nuclear area and more diffuse in cytosolic areas surrounding the nucleus (e.g.: Figure 8A, MBP panel, arrows). The other patterns suggested a structured distribution of MBP, resembling cytosolic filaments organized in a parallel manner, or at the periphery of the cells (e.g.: Figure 8A, MBP panel, arrow heads) and were present only in OL-like cells.

The quantification of the percentage of OL-like cells expressing structured MBP showed that the parallel distribution pattern of MBP could be observed in between approximately 25% and 45% of the cells (Figure 8B, left) and the occurrence of structured MBP at the periphery of the cells (Figure 8B, right) was present in between about 18% and 34% of the cells, depending on the differentiation medium used. Although the combined presence of T3 and F3 seemed to favor the appearance of patterned MBP, we could not find statistically significant differences between the distinct differentiation conditions.

The structured distribution of MBP resembled the pattern of polymerized actin filaments (F-actin). It is known that, in mature oligodendrocytes, MBP associates with F-actin and that this interaction seems to be important for the myelinating activity of OLs [47], [48].

In order to test whether the structured MBP was coincident with polymerized actin filaments, the cells were analyzed for the presence of F-actin using fluorescently-labeled phalloidin. The immunocytochemistry results showed that whenever MBP could be detected in a structured manner (either with the parallel or the peripheral distribution), it always co-localized with F-actin (Figure 8A, merge panel, arrow heads), in contrast to the diffuse MBP, which did not (e.g., Figure 8A, merge panel, arrows). Cells with oligodendrocyte-like morphology could also be observed, displaying co-localization of MBP with F-actin (e.g.: Figure 8A, asterisk).

### Real-time RT-PCR analysis

In order to further characterize the cells obtained at each distinct phase of the differentiation process, cells were analyzed at the transcript level by real-time RT-PCR. *Actin* was used as a house-keeping gene, *fibronectin* as a mesenchymal marker and several neural genes, representative of neural progenitors (*sox2* and *nestin*), neurons (*neurofilament*), astrocytes (*gfap*) and oligodendrocytes (*mbp*), were analyzed.

Data presented in Figure 7E shows that comparing to undifferentiated MSCs, the mesenchymal marker *fibronectin* remained essentially unchanged throughout the differentiation process, while the neural progenitor genes *sox2* and *nestin* were upregulated during the OPC-like stage of differentiation, then returning to lower levels after the OL-like stage. The presence of *nestin* during the OPC differentiation stage of oligodendroglial lineages has been known for some time [41], [42], whereas the expression of *sox2* has only been reported more recently to occur in *bona-fide* OPCs, but not by mature oligodendrocytes [49]–[51]. All other markers did not show any statistically significant difference comparing with MSCs, except for neurofilament, that exhibited a peak at the NSC-like stage and then, in subsequent differentiation steps, decreased to levels similar to those already expressed by MSCs, suggesting that an eventual neuronal-like phenotypic tendency was lost during the later differentiation steps.

Overall, the RT-PCR data seems to be in agreement with the immunocytochemistry results, suggesting that the NSC/OPC-like differentiation stages are associated with the increased expression of neural progenitor markers, while the oligodendroglial maturation marker MBP seemed to be expressed by OL-like cells at levels similar to those already present in undifferentiated MSCs.

### Co-culture experiments

In order to address the interaction between the obtained OL-like cells and neuronal cells, a co-culture experiment was set up [33]. Mouse dorsal root ganglion (DRG) explants were cultured for 10 days until the axons were well developed, and then MSC-derived cells at the end of the OPC-like stage were added to the DRG culture and allowed to be in co-culture for further 14 days in OL-like maturation medium (Materials and Methods).

**Figure 9:**
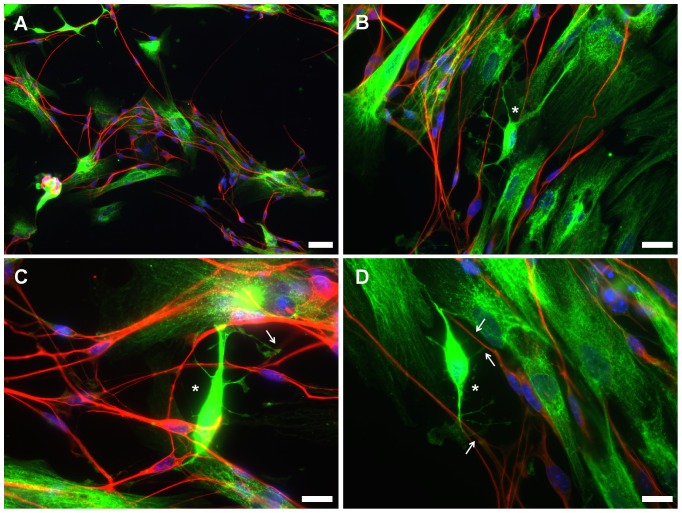
Immunofluorescence microscopy images of OL-like cells derived from hUCM-MSCs in co-culture with mouse dorsal root ganglion (DRG) neurons. The co-cultures were stained using an anti-human Golgi antibody (green) to specifically identify the human cells (OL-like cells derived form MSCs) and an anti-beta-III-tubulin antibody (red) to label the mouse DRG neurons. The images acquired in the green channel (anti-human Golgi antibody) were deliberately slightly overexposed to allow for a better understanding of the cellular morphology, which did not affect the identification of human versus mouse cells, as evidenced by the lack of green signal in the mouse DRG neurons (in red). Counterstaining of the nuclei was performed using DAPI (blue). It was apparent that the OL-like cells and DRG neurons tend to cluster together, as illustrated in a lower magnification image (*A*). Higher magnification images (*B-D*) suggest the existence of contact points (arrows) between branches of OL-like cells displaying an immature oligodendrocyte-like morphology (asterisks) and neurites. Scale bars correspond to 50 µm (*A*) or 20 µm (*B-D*).

By the end of the co-culture period, it could be observed that OL-like cells typically tended to be present in the vicinity of neurons (Figure 9A - neurons stained in red for beta-III-tubulin and OL-like cells stained in green for human Golgi). This observation suggests that there might occur a positive crosstalk between both types of cells (neurons and OL-like cells).

Occasionally, OL-like cells with branched morphology could be observed (Figure 9B-D, asterisk). Although there was no evidence of robust structures resembling myelin sheaths wrapping neurons, in contrast to those described for *bona-fide* oligodendrocytes [33], in some cases there were apparent contact points between branches of OL-like cells and axons of DRGs (Figure 9C, D – arrows). These results suggest that although there was no evidence for robust axonal ensheathment, there might be physical interactions between OL-like cells and neuronal axons.

Overall, the results indicate that MSCs are prone to differentiate into neural-like cells, namely oligodendrocyte-like cells, expressing some of the typical oligodendroglial lineage markers along the distinct differentiation steps. The OL-like cells obtained expressed some of the mature oligodendrocyte markers (e.g.: MBP, GalC), but seemed to be immature comparing to actual *bona-fide* oligodendrocytes in terms of morphology and function. Although some cells did present an OL-like morphology and seemed to establish contacts with axons of neuronal cells, there was no evidence for robust myelination in OL-like/DRG co-culture experiments. Nevertheless, MSCs seem to have the potential to differentiate into oligodendroglial-like lineages and with improvements to the protocol, a more mature phenotype might be attained, with a more conclusive functional phenotype.

## Discussion

Human umbilical cord matrix mesenchymal stem cells (UCM-MSCs) were isolated from 12 umbilical cords with 100% efficiency using a protocol based on that previously described by Reinisch and colleagues [27]. The cells exhibited low generation time and proliferated readily up to at least 8 passages - P8 (Figures 1C-E), reaching a total number of cells over 1×10^9^ after 4 passages, within 17 to 21 days after explant isolation (Figures 1A, B). Nevertheless, it might have been possible to further lower the number of passages and time required to reach this number of cells, which is well above what is considered to be a therapeutic dose of at least 2×10^6^ MSCs/kg of body weight for infusion [29], by increasing the amount of umbilical cord tissue processed, since typically only a small part of each umbilical cord sample was processed. Importantly, the generation time did not increase significantly when cells where passaged until P8, which corresponded to about 20 population doublings (20.6±0.4) - Figure 1C and D -, indicating that the cells did not reach senescence until this number of duplications, in accordance with recent literature that showed that UCM-MSCs could be kept in proliferative conditions *in vitro* until approximately 33 cumulative population doublings (33.7±2.1) before entering replicative senescence [35].

The cells were also able to form colonies with similar frequency (no statistically significant differences were found) at passages 2 and 8 (Figure 1F), further indicating that the cells were proliferative, healthy and maintained stemness for at least 8 passages (or ∼20 population doublings). The efficiency for CFU-F (i.e., the number of colonies formed divided by the total number of cells initially plated) at P2 and P8 was 29.3±4.7 and 26.7±9.9, respectively, comparable to the efficiency described in the literature, of 35.2±2.69 [52]. The slightly higher efficiency reported [52] compared to our study might be explained by the fact that the cell density used in the assay described by Hou *et al.* was substantially higher (50 cells/cm^2^) than the one used in our study (3 cells/cm^2^). By having a higher cell density, we may speculate that the autocrine signaling is favored and a certain threshold to trigger proliferation and colony formation is attained faster, compared to our protocol.

The MSCs obtained from the umbilical cord matrix were able to undergo chondrogenic, osteogenic and adipogenic fates (Figures 2A-C), the typical multilineage differentiation lineages reported for this stem cell type [2]. Nevertheless, we observed low adipogenic induction, a characteristic which has already been reported in the literature for UCM-MSCs, when compared with MSCs from other sources, such as the bone-marrow [53].

The immunophenotypic characterization of the UCM-MSCs obtained further validated their genuine MSC identity, since the cells were positive for CD13, CD73, CD90 and CD105 (Figures 2D-G) and did not expressed CD34, CD45 and CD11b (Figures 2H, K and J), as expected [30], [36], [37]. The presence of nerve growth factor receptor (NGFR, also known as CD271) has been reported in small populations of MSCs from distinct sources, including the bone marrow, umbilical cord matrix and adipose tissue, mostly in fresh samples but not in cultured cells [38], [54], [55]. Regarding the umbilical cord matrix, NGFR has been recently detected to be weakly expressed *in situ* in fresh umbilical cord samples and almost undetected after culture [56]. Since our immunophenotypic analysis was performed only after culture (at P2 and P8), the absence of this marker is therefore not surprising (Figure 2I).

Verifying that the UCM-MSCs isolated were adherent to plastic in culture conditions, presented a typical MSC morphology (Figure 3A and Figure S1) and immunophenotype (Figures 2D-K and Figure S2) and were able to differentiate *in vitro* into osteoblasts, adipocytes and chondroblasts (Figure 2A-C), all the criteria were met [30] to define these cells as *bona-fide* mesenchymal stem cells.

MSCs are plastic stem cells [5] and bone-marrow-derived MSCs were reported to possess, at least partially, a neuroectodermal origin [14], [15]. Moreover, several reports in the literature have shown the potential of MSCs to differentiate into neural-like cells [7], [9], mostly towards neuronal- and astrocyte-like cells [8], [12], [18], [19], while the differentiation of MSCs towards oligodendroglial-like cell types has remained poorly explored (detailed below). Hence, we sought to explore the capacity of UCM-MSCs to differentiate into oligodendroglial-like lineages.

Several reports have shown the induction of pluripotent stem cells (ESCs [24], [25], [39], [57] and iPSCs [26] into oligodendrocyte lineages. Nevertheless, there are concerns regarding the clinical application of cells derived from pluripotent stem cells, like the risk of teratoma formation [58]. Another drawback is low engraftment due to immunological rejection of the transplanted cells by the host immune system, although it was shown that short-term treatments with immunosuppressive drugs in mice enhanced the engraftment of unrelated pluripotent stem cells [59].

Alternatively, MSCs are generally regarded as a safe, non-tumorigenic and low-immunogenic adult stem cell type (with several clinical trials ongoing) [60], that seem to have the potential to differentiate into neural-like cells [7], [9], hence could represent an interesting alternative to pluripotent stem cells for clinical use. The contribution for the establishment of a robust oligodendroglial-like differentiation protocol of MSCs has an important impact for the treatment of demyelinating diseases and regenerative medicine in general.

Few reports have explored the potential of human MSCs to differentiate into oligodendrocyte-like lineages. In one of the first reports, human CD90+ bone-marrow MSCs [21] were differentiated into cells expressing oligodendrocyte markers (O4, NG2, MBP), but also astroglial (GFAP) and, to a lesser extent, neuronal (beta-III-tubulin) markers, leaving unclear what was the true identity of those cells. Nevertheless, some cells seemed to adopt a myelinating oligodendroglial-like behavior in an *in vivo* myelination mouse retina model. Shortly after, Kennea and colleagues [61] obtained cells displaying some of the typical OL-like characteristics by differentiating human MSCs. However, the cells had fetal origin, which represent an ethical, technical and potentially safety drawback. Moreover, the protocol developed required either the use of conditioned medium obtained from the B104 rat neuroblastoma cell line or the overexpression of the pro-oligodendrocyte Olig-2 gene delivered by lentiviral transduction, further raising difficulties for possible future therapeutic applications. More recently, UCM-MSCs were differentiated into OL-like cells [22], however this study focused mostly on the secretome and neurotrophic effect of the oligodendrocyte progenitor-like cells obtained.

Taking into account several protocols available in the literature describing neural-like and oligodendroglial-like lineage induction of distinct stem cell types [7], [9], [22], [24], [39], [57], we designed and optimized a step-wise protocol, testing distinct media, culture substrates and coating conditions for this endeavor (Figure S3).

Undifferentiated MSCs expressed nestin to a certain extent [11] (Figure 3E) and the presence of bFGF and EGF was reported to enhance the expression of this neural marker and confer cells with a neural precursor-like phenotype, as observed by the formation of neurosphere-like structures when cultured in low attachment conditions [9]. We used a similar approach (data not shown), but in parallel to the neurosphere-like approach also adopted by Zhang and colleagues for the differentiation of MSCs into OL-like cells [22], we decided to test a 2D approach (cells in monolayer), similar to what had been previously reported to obtain neural progenitors from ESCs [62]. Our approach was carried out using non-treated (non-tissue culture) polystyrene plates, that conferred some degree of attachment to the cells, but due to low adhesiveness, promoted the formation of compact 2D cell clusters or colonies that expressed high levels of nestin (Figures 3C and F). This allowed for a simpler culture system and avoided the difficulties of dissociating the 3D cell clusters, which resulted in considerable cell death. Moreover, during culture, substantial cell death occurred in the interior of the neurosphere-like structures due to the difficulty in controlling the sphere size (not shown), probably due to limited access of nutrients to those regions.

Compared to undifferentiated MSCs, NSC-like cells were strongly positive for the neural stem/progenitor marker nestin (Figures 3E and F, 4A and D and 7A). In this stage of differentiation, cells were essentially negative for OPC markers such as A2B5 and O4 (Figures 4E, F and 7B). Surprisingly, there was an increase in the expression of MBP and GalC during this differentiation step (Figures 7C and D), which may be a result of the fact that undifferentiated MSCs already expressed a basal level of MBP and GalC (Figures 6S and T and Figures 7C and D), in agreement with previous reports [7]. NSC-like cells also seemed to express other neural markers, such as β-III-tubulin and GFAP (data not shown) or neurofilament, as addressed by RT-PCR (Figure 7E). Again, in agreement with our observations (Figures 5J and L), previous reports stated that some undifferentiated MSCs expressed such neural markers [7], which may explain the observation of β-III-tubulin and GFAP during the neural-like commitment of the cells. Nevertheless, these neuronal and astrocytic markers were essentially absent or downregulated in the last stage of differentiation, especially in cells that were cultured on fibronectin during the OPC-like stage of differentiation (Figures 5A-H and Figure 7E).

During the OPC-like stage of differentiation, cells acquired a bipolar morphology and typical oligodendrocyte precursor lineage markers such as A2B5 and O4 (Figure 4). Moreover, the presence of the neural stem/progenitor marker nestin at both the protein (Figure 4) and mRNA level (Figure 7E) were consistent with the oligodendrocyte precursor-like stage of these cells [41], [42]. However, the profile of mRNA and protein expression of nestin did not overlap completely. While the peak of nestin protein expression occurred at the NSC-like stage (Figure 7A), the peak of *nestin* mRNA happened at the OPC-like phase (Figure 7E). One hypothesis that can be considered to explain such result is the presence of PDGF in the culture media during the OPC-like induction step of the differentiation protocol, a growth factor that was shown to induce transcription of *nestin* [42]. Moreover, the levels of nestin protein were reported to be controlled at a post-translational level, namely through proteasome-mediated degradation upon differentiation of NSCs [63], which may account for the lower protein level of nestin in OPC-like cells compared with NSC-like cells despite the mRNA levels being higher in the first. Along with *nestin*, *sox2* was also upregulated during the OPC-like stage (Figure 7E) and although this marker was typically considered to be absent in OPCs [41], the expression of sox2 was very recently reported in oligodendroglial progenitors in the spinal cord of mice and rats by at least two independent research groups [49]–[51]. Interestingly, sox2 was reported as a possible regulator of *nestin* [64], which may explain the similar expression profile of both genes (Figure 7E).

The presence of ECM proteins like merosin (previously used by Zhang *et al*. [22]) or fibronectin seem to have favored equally well the survival of OPC-like cells, in contrast with uncoated dishes, in which very substantial cell loss was observed (Figure 4). Nevertheless, it became apparent during the OL-like stage of differentiation that the presence of FN during the OPC-like stage resulted in a subsequent more evident decrease of the OPC marker A2B5 in the OL-like cells, in comparison to OPC-like cells cultured in presence of merosin (Figure 6U and V), suggesting a higher maturation of the OL-like cells that were previously cultured in the presence of FN during the OPC-like stage.

Moreover, the OL-like cells obtained expressed typical oligodendrocyte differentiation markers. O4 and GalC (in case of differentiation in presence of T3 or F3), were upregulated in OL-like cells, compared to undifferentiated MSCs (Figures 7B and C). O4 is present throughout several stages of differentiation of *bona-fide* oligodendrocytes, namely from the OPC to the mature OL stage, whereas GalC is present only in differentiated OLs, although this marker can be detected in both immature and mature differentiated oligodendrocytes [46], [65]. On the other hand, the OL-like cells obtained exhibited only low amounts of MBP (Figure 7D), a marker that is typically present only in mature OLs [46], [65]. Taken together, these results suggest that the OL-like cells obtained have a differentiated, but not fully matured OL-like phenotype.

Despite the immature phenotype, several OL-like cells displayed a patterned MBP distribution that was consistent with F-actin co-localization, unlike that of MSCs (Figure 8). This co-localization pattern is similar to the co-localization that has been reported for *bona-fide* oligodendrocytes [47], [48].

Moreover, co-culture experiments showed that OL-like cells were typically in close vicinity of neurons, suggesting that a positive crosstalk between both cell types might be present. The nature of such interactions may depend on soluble factors and/or direct cell-cell contact, although further experiments would be required to address that specific issue. It was also evident that some of the OL-like cells displayed a branched oligodendrocyte-like morphology (Figure 9), that suggested the existence of contact points between both cell types. Nevertheless, no clear signs of axonal ensheathment by OL-like cells were visible.

In summary, we have shown that cells isolated from the umbilical cord matrix are *bona-fide* mesenchymal stem cells. We have confirmed the neural-like plasticity of MSCs and explored their oligodendroglial-like commitment using a step-wise differentiation protocol. Cells displayed several neural and oligodendroglial markers at specific points of the differentiation protocol, suggesting an oligodendroglial-like specification of the cells along the process. Although a fully differentiated phenotype has not been reached, some typical and prominent features were similar to those of *bona-fide* oligodendrocytes.

## Supporting Information

Figure S1 **Isolation of MSCs from umbilical cord matrix explants.** Proliferating MSCs with a fibroblastoid-like shape could be readily identified migrating from umbilical cord matrix fragments after a 10 days culture period in proliferation medium (see Materials and methods). Scale bar corresponds to 200 µm.
(TIFF)

Figure S2 **Immunophenotype of UCM-MSCs.** Immunophenotypic characterization by flow cytometry of three independent donor samples - UCM#2 at passage 2 (*A*) and P8 (*B*), UCM#3 at P2 (*C*) and P8 (*D*), and UCM#7 at P2 (*E*) and P8 (*F*).
(TIFF)

Figure S3 **Overview of the experimental conditions tested to differentiate hUCM-MSCs into oligodendrocyte (OL)-like cells.** Schematics of the differentiation protocol through the different stages (S0 to S4), and respective nomenclature. Soluble factors (Sol. Factors), surfaces and alternative coatings used at the distinct steps of differentiation are indicated.
(TIFF)

Figure S4 **Assessment of the expression of MBP in hUCM-MSCs by western-blot analysis.** Western-blot analysis was performed using antibodies against MBP and GAPDH (as loading control). MBP could be readily detected (band at ∼33 kDa, as announced by the manufacturer of the antibody) in protein extracts of MSCs (35 µg of total protein per lane) and rat brain cortex extracts (15 µg of total protein per lane), the latter being used as a positive control (*A*). GAPDH was used as a reference protein to calculate the relative expression of MBP, based on the ratio of the integrated densities of the band of MBP divided by that of GAPDH, for each sample (*B*). It could be observed that despite expressing much less MBP than that found in rat brain cortex, MSCs expressed appreciable levels of MBP.
(TIFF)

Table S1 **Figure 1** **data.** Raw data used to produce Figure 1.
(XLS)

Table S2 **Figure 7** **data.** Raw data of MFI values used to produce Figure 7.
(XLS)

Table S3 **Figure 8** **data.** Raw data used to produce Figure 8B.
(XLS)
