# Supplementary figures and images for: Differentiation of Human Umbilical Cord Matrix Mesenchymal Stem Cells into Neural-Like Progenitor Cells and Maturation into an Oligodendroglial-Like Lineage
Source: PLoS One. 2014 Oct 30;9(10):e111059. doi: 10.1371/journal.pone.0111059 (PMC4214693; doi:10.1371/journal.pone.0111059)

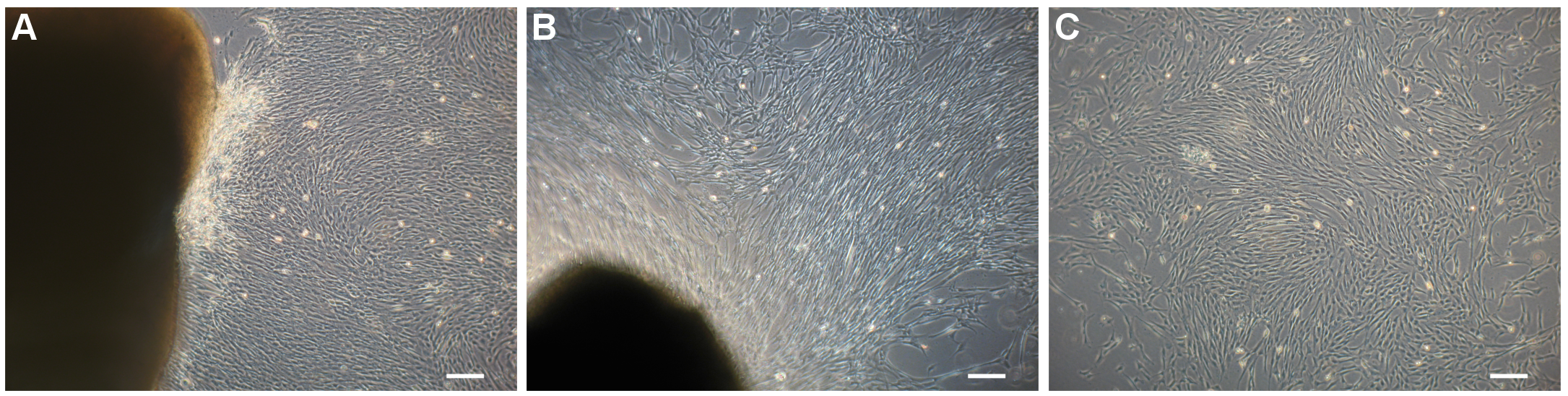

Supplement: Figure S1 — Isolation of MSCs from umbilical cord matrix explants. Proliferating MSCs with a fibroblastoid-like shape could be readily identified migrating from umbilical cord matrix fragments after a 10 days culture period in proliferation medium (see Materials and methods). Scale bar corresponds to 200 µm. (TIFF) [file pone.0111059.s001.tiff]

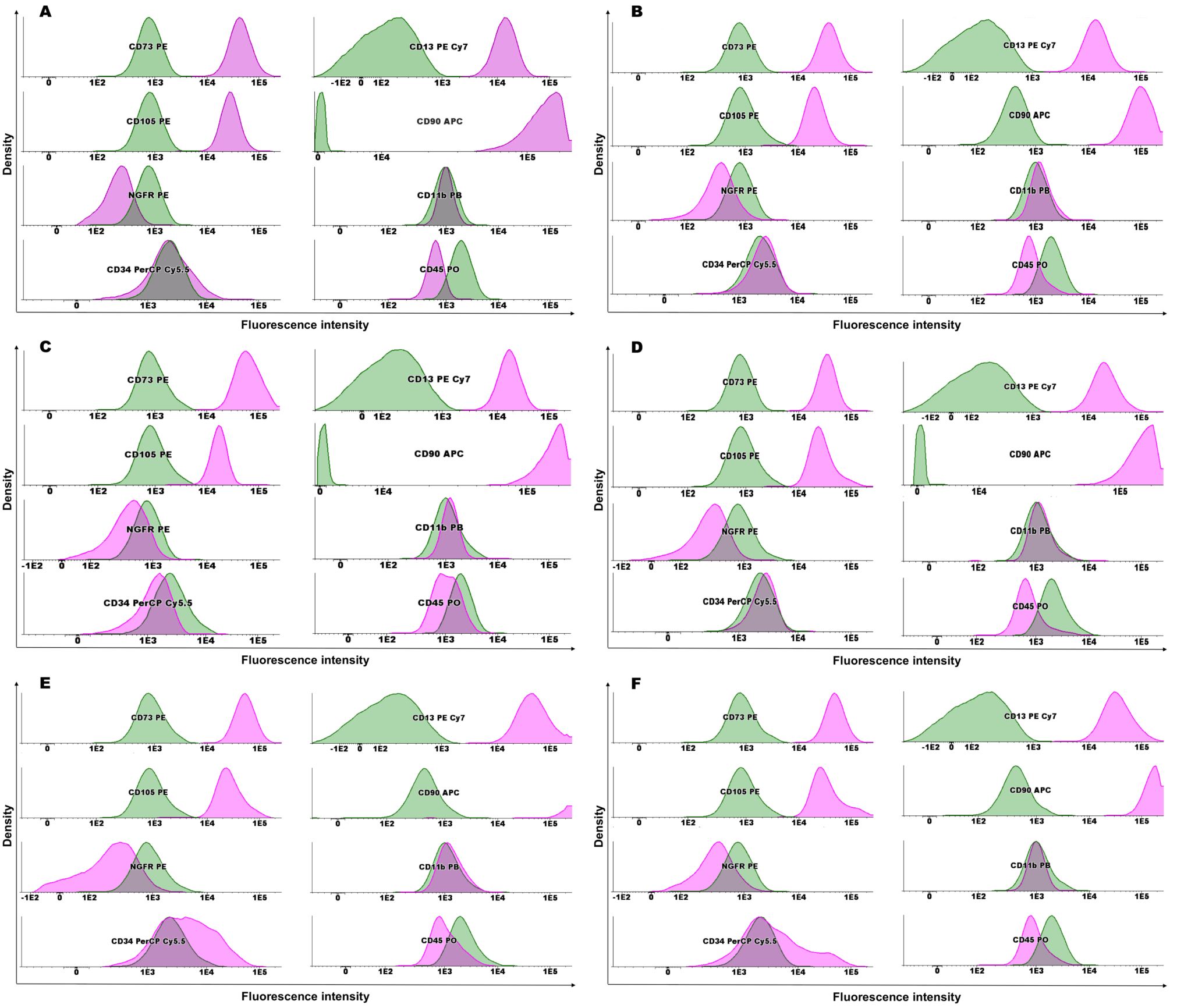

Supplement: Figure S2 — Immunophenotype of UCM-MSCs. Immunophenotypic characterization by flow cytometry of three independent donor samples - UCM#2 at passage 2 (A) and P8 (B), UCM#3 at P2 (C) and P8 (D), and UCM#7 at P2 (E) and P8 (F). (TIFF) [file pone.0111059.s002.tiff]

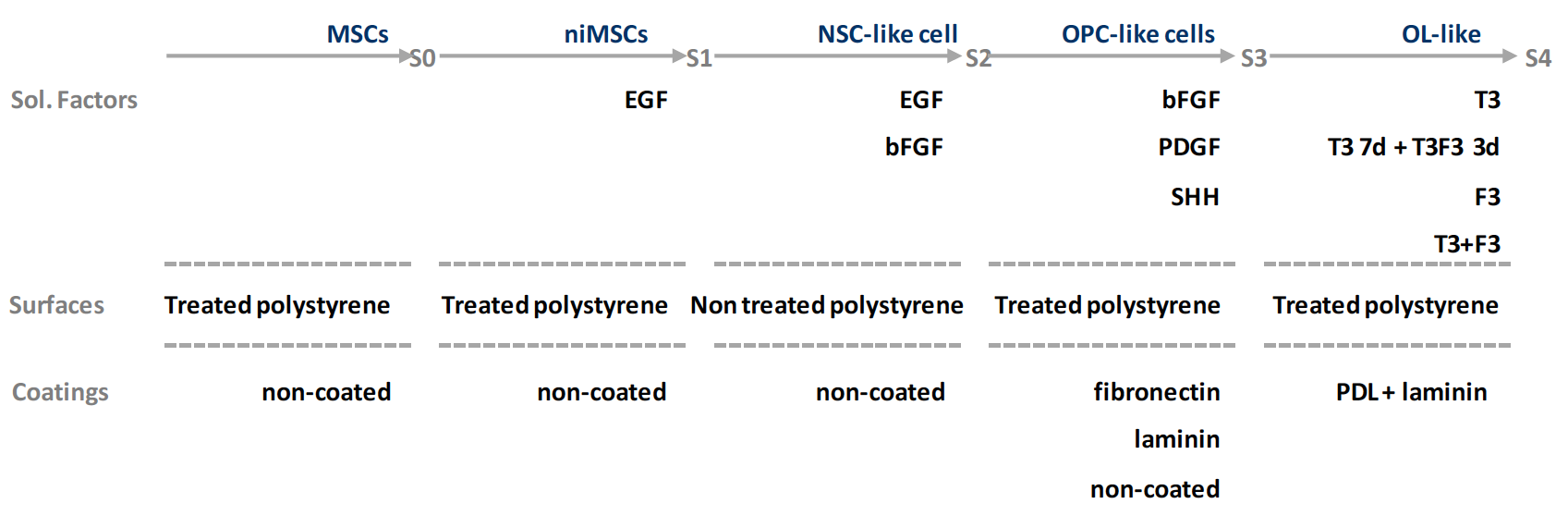

Supplement: Figure S3 — Overview of the experimental conditions tested to differentiate hUCM-MSCs into oligodendrocyte (OL)-like cells. Schematics of the differentiation protocol through the different stages (S0 to S4), and respective nomenclature. Soluble factors (Sol. Factors), surfaces and alternative coatings used at the distinct steps of differentiation are indicated. (TIFF) [file pone.0111059.s003.tiff]

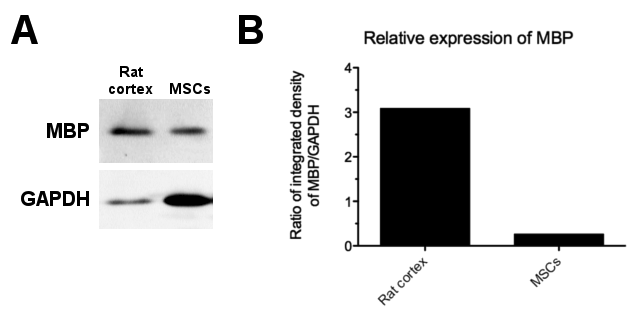

Supplement: Figure S4 — Assessment of the expression of MBP in hUCM-MSCs by western-blot analysis. Western-blot analysis was performed using antibodies against MBP and GAPDH (as loading control). MBP could be readily detected (band at ∼33 kDa, as announced by the manufacturer of the antibody) in protein extracts of MSCs (35 µg of total protein per lane) and rat brain cortex extracts (15 µg of total protein per lane), the latter being used as a positive control (A). GAPDH was used as a reference protein to calculate the relative expression of MBP, based on the ratio of the integrated densities of the band of MBP divided by that of GAPDH, for each sample (B). It could be observed that despite expressing much less MBP than that found in rat brain cortex, MSCs expressed appreciable levels of MBP. (TIFF) [file pone.0111059.s004.tiff]
